# A scoping review of therapeutic reasoning process research

**DOI:** 10.1007/s10459-022-10187-7

**Published:** 2023-04-12

**Authors:** Quang Hung Duong, To Nhu Pham, Lorenna Reynolds, Yan Yeap, Steven Walker, Kayley Lyons

**Affiliations:** 1 Faculty of Pharmacy and Pharmaceutical Sciences, Monash University, 381 Royal Pde, Parkville, 3052 Australia; 2 Centre for Digital Transformation of Health, University of Melbourne, 700 Swanston St, Carlton, 3053 Australia

**Keywords:** Learning theories, Scoping review, Therapeutic decision-making, Therapeutic reasoning, Decision making processes

## Abstract

Therapeutic reasoning is when the purpose, task, or goal for engaging in reasoning is to determine the patient’s management plan. As the field’s understanding of the process of therapeutic reasoning is less well understood, we focused on studies that collected data on the process of therapeutic reasoning. To synthesize previous studies of therapeutic reasoning characteristics, methodological approaches, theoretical underpinnings, and results. We conducted a scoping review with systematic searching for English language articles with no date limits. Databases included MEDLINE, CINAHL Plus, Scopus, Embase, Proquest Dissertations and Theses Global, and ERIC. Search terms captured therapeutic reasoning in health professions education research. Initial search yielded 5450 articles. The title and abstract screening yielded 180 articles. After full-text review, 87 studies were included in this review. Articles were excluded if they were outside health professions education, did not collect data on the process of therapeutic reasoning, were not empirical studies, or not focused on therapeutic reasoning. We analyzed the included articles according to scoping questions using qualitative content analysis. 87 articles dated from 1987 to 2019 were included. Several study designs were employed including think-aloud protocol, interview and written documentation. More than half of the articles analyzed the data using qualitative coding. Authors often utilized several middle-range theories to explain therapeutic reasoning processes. The hypothetico-deductive model was most frequently mentioned. The included articles rarely built off the results from previous studies. Six key result categories were found: identifying themes, characterizing and testing previous local theory, exploring factors, developing new local theory, testing tools, and testing hypothesis. Despite the cast body of therapeutic reasoning research, individual study results remain isolated from previous studies. Our future recommendations include synthesizing pre-existing models, developing novel methodologies, and investigating other aspects of therapeutic reasoning.

**Supplementary Information:**

The online version contains supplementary material available at 10.1007/s10459-022-10187-7.

## Introduction

Proficient clinical reasoning is considered a core competency for health professionals and a central tenet of sound patient-centered therapeutic decision-making (Meredith Young et al., 2019). Clinical reasoning includes both the thinking processes for how health professionals make decisions regarding diagnoses (i.e., diagnostic reasoning) and therapies (i.e., therapeutic reasoning). In health care, therapeutic reasoning underpins a key role of physicians, pharmacists, nurses, and more; ensuring that therapies are safe, effective, ethical and patient-centered (Baker et al., 2017; Durning et al., 2011a; Myers et al., 2017; Thackray & Roberts, 2017). Therefore, it is an essential aspect of healthcare professional training to implement strategies to strengthen therapeutic reasoning skills of learners (Eva, 2005; Rencic, 2011; Meredith Young et al., 2018).

Clinical reasoning comprises both “diagnostic reasoning” (i.e., formulate a correct diagnosis) and “therapeutic reasoning” (i.e., formulate the best possible treatment plan) (Durning et al., 2011a). In comparison to diagnostic reasoning, therapeutic reasoning is relatively understudied and less well understood (Cook et al., 2018a, 2019; Norman, 2005). For diagnostic reasoning, there are several reviews on how diagnostic reasoning occurs, should be taught, and could be assessed (Custers et al., 1996; Daniel et al., 2019; Gruppen, 2017). Also, several authors have reviewed and explored what defines and theoretically underpins diagnostic reasoning (Cook et al., 2019; Croskerry, 2009; Norman, 2005; Schmidt & Rikers, 2007). In contrast, there are no exclusive reviews of therapeutic reasoning literature (Cook et al., 2019).

Cook et al. (2019) have proposed six research priorities for therapeutic reasoning. This review maps the relevant literature to facilitate addressing the following two research priorities; (1) to develop and use new research paradigms and techniques, and (2) to understand the cognitive processes underlying therapeutic reasoning. Processes include the reasoners’ steps, strategies, components, and rationale they used to solve the problem (Meredith Young et al., 2019). Currently, there are several published studies to explore practitioners’ therapeutic reasoning processes (Abuzour, 2017; Wright et al., 2019) (e.g., Bartels, 2013; Croft et al., 2018; Nusair, 2017), but their methods, theories, and results have not been synthesized. Presumably, this literature body has not yet been reviewed as it contains many dissertations, articles in several disciplines (e.g., nursing, medicine, pharmacy, occupational therapy, physical therapy), and inconsistent terminology (Young et al., 2019). For example, many studies use the term “clinical reasoning” when they are solely focused on “diagnostic reasoning” and not therapy, particularly in medicine. Very recently, a paper attempted mapping the available terminologies on clinical reasoning (Young et al., 2020). The researchers identified the multifaceted and complex nature of clinical reasoning. However, they did not limit their findings to therapeutic reasoning, nor did they investigate further into how clinical reasoning manifested in these professions. It is acknowledged that while therapeutic reasoning might differ from clinical reasoning (or rather, diagnostic reasoning) in medicine, is more aligned to the term “clinical reasoning” in other health professions, such as nursing or physiotherapy (Young et al., 2020). The terms “clinical reasoning”, “management reasoning”, “intervention reasoning”, and “treatment decision making” in nursing and rehabilitation professions all resemble closely the term “therapeutic reasoning” in this review, focusing on devising treatment plans, rather than making diagnoses.

Before researchers can move forward with Cook et al.’ (2018b; Cook et al., 2019) call for further research on processes, it would be beneficial to synthesize previous studies on therapeutic reasoning processes. Specifically, future researchers would benefit from an understanding of previous studies’ settings, populations, methodological approaches and results.

Most important of all, a synthesis of previous theoretical underpinnings and conceptual frameworks would advance this literature and research area. As in most health professions education research, there is immense vastness and variability in theoretical frameworks. Future researchers and educators would benefit from a mapping of middle-range and local theories of therapeutic reasoning. Middle-range theories are theories that were developed from multiple studies and could apply to processes beyond therapeutic reasoning processes (e.g., dual-process theory) (McKenney & Reeves, 2013). In contrast to middle-range theories, local theories are understandings that originated from specific investigations and only applied to therapeutic reasoning (McKenney & Reeves, 2013). By mapping these theories across the literature, future researchers will be better informed about what theories could apply to their investigations and build off gaps in the literature.

As a result, we undertook this scoping review to (1) investigate the nature of research activity, explore theoretical understandings, summarize research findings, identify gaps in the therapeutic reasoning literature and (2) synthesize proposed theoretical reasoning models frameworks. By summarizing the nature and findings of previous research, this review can aid future researchers and educators interested in therapeutic reasoning processes.

Specifically, our scoping questions included:

1. What are the characteristics of published articles investigating the process of therapeutic reasoning?
2. What study methods have authors used to study the process of therapeutic reasoning?
3. What middle-range theories do authors use to position therapeutic reasoning?
4. What local theories do authors employ to explain the processes of therapeutic reasoning?
5. What are the key results of studies that investigate therapeutic reasoning?

## Methods

To answer our research questions, we conducted a scoping review (Peters et al., 2015). We were interested in summarizing the answers to our scoping questions, identifying areas of strength and gaps in the literature. Whereas systematic reviews are used to investigate discrete quantitative questions, a scoping review is used to map answers to key questions in a literature with a variety of study methods.

### Definitions

Young and colleagues have recently published a review to distinguish between terminologies in clinical reasoning (Meredith Young et al., 2019). This framework was used by the research team to distinguish between concepts in the literature. Only studies that involved therapeutic reasoning processes were included in the study. We adapted ideas in the Young and colleagues manuscript to define therapeutic reasoning as “when the purpose, task, or goal for engaging in reasoning is to determine the patient’s therapeutic management plan”. Reasoning processes are defined by Young and colleagues: process components of clinical reasoning, encompassing processes, strategies, components, steps, or rationale used to solve the problem. Terms reflecting reasoning processes tended to be tightly linked to a variety of cognitive or psychology-based frameworks (Meredith Young et al., 2019).

### Search strategy

Our systematic search strategy was determined through consultation with the University librarian, and adjusted according to response and increasing familiarity with the topic during the search process. Health science and education databases, and selected journals, were searched independently as described below (“database search” and “journal keyword search”, respectively).

### Database and journal search

The database search strategy is reported in Table 1. The databases searched were MEDLINE, CINAHL Plus, Scopus, Embase, and ERIC. Synonymous terms to “therapeutic reasoning” were “therapeutic management,” “management reasoning,” therapeutic decision making,” “clinical reasoning,” and “clinical decision making.” All search terms were first piloted in MEDLINE database to balance sensitivity and specificity in the search. The topic terms were cross-searched with a list of common health professional terms (e.g., “physiotherapist”)as shown in Table 1 and terms related to training (“student,” “practitioner,” “expert,” “resident,” “intern,” and “registrar”). The training search terms were cross-searched with the health professional and topic terms to decrease the false negatives from practice research (e.g., clinical trials). The example in Table 1 demonstrates the use of phrase searching, boolean operators, truncation, wildcards, and proximity operators in the search. Advanced search techniques (e.g. wildcards, truncations) were adapted to each individual database. MEDLINE search also included related subject headings. Searches were limited to the English language. Gray literature was searched using the search terms in the following databases: dissertations and theses in Proquest Dissertations and Theses Global database and 2) book chapters, books, and conference papers in MEDLINE, CINAHL Plus, Scopus, Embase, and ERIC. Gray literature was included specifically to enrich the analysis and to address publication bias, as it was believed that peer-reviewed therapeutic reasoning research was lacking.

**Table 1:** Database search strategy (example reported here was used for CINAHL Plus)

["therapeutic reason*" OR "therapeutic manag*" OR "management reason*" OR "therapeutic decision making" OR "clinical reason*" OR "clinical decision making"]	
AND	
["pharmacist*" OR "physician*" OR "occupational therapist*" OR "nurse*" OR "physical therapist*" OR "physiotherapist*" OR "dentist*" OR "dieti#ian*" OR "nutritionist*" OR "podiatrist*" OR "optometrist*" OR "allied health practitioner*" OR "allied health professional*" OR "midwi#e*" OR "speech pathologist*" OR "psychologist*" OR "psychiatrist*" OR "paramedic*" OR "audiologist"]	
AND	
["student*" OR "practitioner*" OR "expert*" OR "resident*" OR "intern*" OR "registrar*"]	

NB: A keyword search of “clinical decision making” in Embase returned too many results (> 5000), therefore “clinical decision making” adj20 process was performed as a title search instead

Since not all health professions journals are indexed in databases, we conducted a journal keyword search. Selected journals from ‘Annotated Bibliography of Journals for Educational Scholarship (AAMC-Regional Groups on Educational Affairs)’ were searched manually using the terms "therapeutic reasoning", "therapeutic management", "management reasoning" and "therapeutic decision making". Each term was searched separately. The databases and journals were searched over a span of six weeks (29 August—9 September 2019 for the keyword journal search; 4 October 2019 for the database search).

### Study screening

All studies and bibliographic information were downloaded using EndNote X9 (Clarivate Analytics, Philadelphia, PA, USA). Duplicate removal and article screening were also completed using EndNote X9 software. In total, 7358 citations were retrieved, from which 1908 duplicates were removed, resulting in 5450 articles to be screened.

The 5450 articles were independently screened according to the inclusion and exclusion criteria (Table 2). Studies were included if they met the following criteria: (1) any date, (2) English language, (3) the main focus of the study is therapeutic reasoning, (4) the article type was either a peer-reviewed journal article, book, book chapter, dissertation, theses, or published conference paper, (5) it was an empirical study collecting data on the reasoning process (as opposed to outcomes or context), and (6) the research subjects were health professionals and learners.

**Table 2:** Inclusion and exclusion criteria

*Inclusion criteria*	
· Any date	
· English language	
· Main topic: therapeutic reasoning or synonymous term	
· Article type: peer-reviewed journal article, book, book chapter, dissertation/theses or published conference paper	
· Study method: empirical studies that collect data on the reasoning process. For example:	
o Think-aloud	
o Observations	
o Interviews, focus groups or surveys post-stimulus (e.g. post case study)	
o Stimulus tests (e.g. script concordance tests)	
· Subject: pharmacist, nurse, doctor, dentist, occupational therapist, physiotherapist, paramedic, midwife, dietitian/nutritionist, psychiatrist, psychologist, audiologist, speech pathologist, allied health professional	
*Exclusion criteria*	
· Non-English language	
· Other type of reasoning (e.g. diagnostic reasoning, decisions to admit or discharge patients from hospital)	
· Article type: expert opinion/commentary, literature/systematic reviews	
· Study method:	
o Non-empirical studies	
o Does not collect data on the reasoning process. For example:	
▪ Nationwide surveys	
▪ Instrument development	
▪ Interviews, focus groups or surveys collected at one point in time that has no actual stimulus (e.g. case study)	
▪ Treatment guidelines	
▪ Clinical drug trials	
Subject: pre-professional (i.e. yet to begin undergraduate healthcare degree)	

**Fig. 1:**
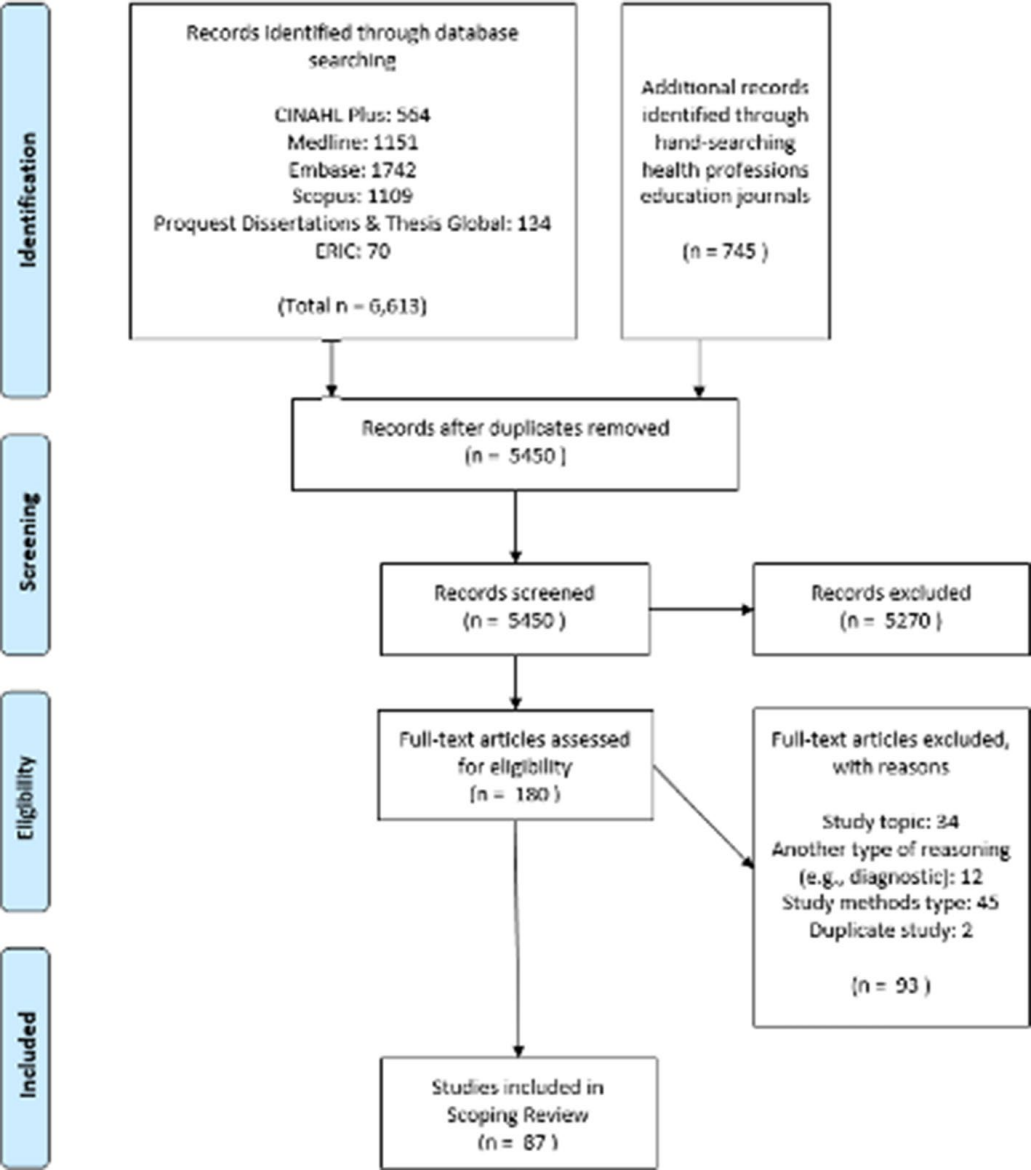
PRISMA (preferred reporting items for systematic reviews and meta-analyses) flow diagram of article selection

The screening and appraisal procedure were performed stepwise, with titles being screened first, followed by abstracts, and finally full texts. KL, YY and LR met prior to the title screen and throughout the process to ensure agreeance on the inclusion and exclusion criteria. Each abstract and full-text article was screened by two researchers (YY and LR). The lead researcher (KL) resolved any discrepancies after consultation. Concluding this process, the articles remaining for extraction were 87 (Fig. 1).

### Data extraction

A synthesis matrix was created to help organize the data. All included articles (n = 87) were then read in full by two authors (NP and HD) to conduct an in-depth data extraction. We extracted both general and specific information from each study, as suggested by Arksey and O'Malley (2005). General information included authors, years of publication, journal names and study locations. Study-specific information consisted of professions, methodological approaches, participant types, data analysis methods, settings, underpinning theoretical models (middle-range and local theories only), research questions and summary of results. NP and HD performed data extraction independently. These authors then crosschecked each other’s work and resolved discrepancies. The matrix was also reviewed by the lead author (KL) to ensure accuracy of data extraction.

### Content analysis

After the extraction, charting and review of data, three authors (KL, NP and HD) inductively generated a list of codes to characterize the included studies according to our scoping questions. The majority of codes were simple in their definitions. For example, to examine what types of methods authors used, we coded data collection as either “interviews,” “think-aloud protocol,” or “written documentation.”

The scoping questions related to middle-range and local theory required discrete definitions. Both middle-range and local theories may be used to describe, explain, and predict therapeutic reasoning processes. We defined middle-range theories as theories that were developed from multiple studies and could apply to processes beyond therapeutic reasoning processes (e.g., dual-process theory) (McKenney & Reeves, 2013). To avoid misinterpreting the authors’ work, we only coded for middle-range theories if the author explicitly referred to and cited the theory. In contrast to middle-range theories, local theories were understandings that originated from specific investigations and only applied to therapeutic reasoning (McKenney & Reeves, 2013). We recognize that others may define middle-range and local theories as models, frameworks, underpinnings, paradigms, and more.

Overall, the distinction between high-level, middle-range, and local theories is not exact, but can be viewed on a continuum (McKenney & Reeves, 2013). However, similar to many authors, we decided to categorize theories according to this distinction to achieve our research aims. High-level theories incorporate multiple middle-range theories (McKenney & Reeves, 2013). In most cases, it is not necessary for authors to state which high-level theory they used as middle-range theories can be mapped to these higher-level theories. After extracting the names of middle-range theories, we categorized them into the following three high-level general categories using references (Schunk, 2016) and our knowledge of theory: cognitive, metacognitive, and contextual. Then we created a visual map of the categories, theories, and some key concepts in these theories to demonstrate to the reader the landscape of middle-range theories in therapeutic reasoning.

We followed content analysis procedures (Hsieh & Shannon, 2005). The authors conducted an initial face-to-face moderation trial to code a sample of extracted data. This process involved rereading each study by paragraph, charting our interpretations of the results and manually categorizing the extracted data to unravel the key context and purposes. The remaining data were then coded independently and iteratively by two authors (NP and HD), with extensive post-analysis discussion to achieve consistency and reliability. We collated these codes to establish themes and subthemes, which were refined, defined and finalized.

## Results

### What are the characteristics of published articles investigating the process of therapeutic reasoning?

The 87 included studies dated from 1987 to 2019 (Table 3). They were conducted in 12 different countries: 42 from the USA (46%), 16 from UK (18%), 14 from Oceania (15%), 11 from Canada (12%), 7 from Europe (8%), 3 from Asia (3%), and 1 from Africa (1%). There were 74 journal articles (82%), 14 dissertations (15%), and 3 other publications (3%). Four main professions were represented across the studies including nursing, medicine, physical therapy and occupational therapy. Dentistry, optometry, pharmacy, and other allied health professions were examined to a lesser extent. We classify the participants into two levels, which are beginners (i.e., students, interns or residents), and advanced professionals. 56 studies investigated advanced professionals, 27 investigated beginners (e.g., undergraduates, interns and residents practitioners), and 8 investigated both levels.

**Table 3:** Demographics of included articles

Characteristics	Categories	Number of studies (%) N = 87 articles
Year	1985 – 1989	2 (2.3)
	1990 – 1994	6 (6.9)
	1995 – 1999	9 (10.3)
	2000 – 2004	12 (13.8)
	2005 – 2009	7 (8.1)
	2010 – 2014	19 (21.8)
	2015 – 2019	32 (36.8)
Profession ^a^	Nursing	27 (32)
	Medicine	24 (26)
	Physical therapy	15 (18)
	Occupational therapy	10 (11)
	Pharmacy	5 (5)
	Dentistry	2 (2)
	Optometry	1 (1)
	Other ^b^	7 (9)
Study location ^c^	USA	40 (46)
	UK	15 (17.2)
	Australia	12 (13.8)
	Canada	11 (12.6)
	Sweden	3 (3.5)
	Hong Kong	2 (2.3)
	Netherlands	2 (2.3)
	New Zealand	2 (2.3)
	Norway	1 (1.2)
	Kenya	1 (1.2)
	Korea	1 (1.2)
	Switzerland	1 (1.2)
Participant	Beginners ^d^	26 (29.9)
	Advanced professionals	53 (60.9)
	Both levels	8 (9.2)

^a^ Some studies may include more than one profession. The percentage adds up to more than 100 percent

^b^ This includes social work, podiatry, education play therapy, music therapy, prosthetics, speech pathology, psychiatry, psychology

^c^ Some studies may be conducted at multiple locations. The percentage adds up to more than 100 percent

^d^ Beginners include undergraduates, interns and residents practitioners

### What study methods have authors used to study the process of therapeutic reasoning?

To measure therapeutic reasoning, researchers provided participants with a stimulus in a setting that enabled the manifestation of the therapeutic reasoning process. The most common stimulus was patient cases, which were either mock experience (n = 52) or real-life patient encounters (n = 22). The mock experience encompassed paper-based written cases, multiple choice questions, physical simulations and digital simulations. Video-taped clinical interactions were also employed to stimulate therapeutic reasoning (n = 5). Questions were used as a stimulus for semi-structured interviews, structured discussions, and written documentation (n = 13).

Authors captured therapeutic reasoning processes using a variety of methods. Most commonly, authors conducted interviews (n = 33), think-aloud protocol (n = 37), or written documentation (n = 22). The think-aloud protocol, a process that participants verbalize their thoughts while performing a task, was employed concurrently (i.e., happening during stimulus) and retrospectively (i.e., happening after the stimulus) (Ericsson & Simon, 1980). Written documentation also included either concurrent or retrospective surveys (Brennan & Spencer, 2002; Crowe, 2012; Myers et al., 2017) written reflections (Nilsson & Lindström, 2016), field diaries (Mancuso & Rose, 1987) or discussion forums (Fiddler et al., 2016).

Qualitative coding was a frequent approach to analyzing therapeutic reasoning processes (n = 65). More than half of the studies (n = 48) inductively coded raw data, generating their own codes and themes (Schadewitz & Jachna, 2007). A considerable number of researchers utilized other types of coding, namely “semi-inductive coding” (Fereday & Muir-Cochrane, 2006) (n = 7) and “deductive coding” (Schadewitz & Jachna, 2007) (n = 10). These researchers referred to and incorporated, in parts or entirely, components of past models into their coding framework. Although in most articles the underlying theories for coding were explicitly stated, three articles stated they consulted literature for analysis but did not refer to any specific theories or frameworks (Brennan & Spencer, 2002; Faucher et al., 2012; Orme & Maggs, 1993).

In addition to coding, novel and emerging methodologies were utilized by a few articles (n = 6) to investigate therapeutic reasoning processes. This included magnetic resonance imaging (Chang et al., 2016; Durning et al., 2012b), gallery walk (Cristancho et al., 2017), laddering (Michael Curran et al., 2006b) and narrative analysis (Baker et al., 2017; Lee & Ryan-Wenger, 1997). Interestingly, one study asked participants to draw pictures to visualize the reasoning process (Cristancho et al., 2017). Additionally, researchers also analyzed the process via their own observation (n = 7), but always in combination with other methods. In some instances, quantitative methodology was employed to measure therapeutic reasoning (n = 14), sometimes complementary to the qualitative methods. These studies often compared therapeutic reasoning between two groups, or calculated frequency of therapeutic reasoning steps. Others (n = 8) assessed participants using a standardized rubric or scoring instrument.

### What middle-range theories do authors use to position therapeutic reasoning?

Table 4 lists the local theories and middle-range theories cited by included articles. Middle-range theories are theories that were developed from multiple studies and could apply to cognitive processes beyond therapeutic reasoning processes (e.g., dual-process theory) (McKenney & Reeves, 2013). Collectively, 28 studies underpinned their studies with a middle-range theory. The hypothetico-deductive model (Elstein et al., 1990) was used most often (n = 7) and commonly adopted in research on non-expert or non-medical participants. The second most implemented theory was the information processing theory (Newell, 1972) (n = 4). Other theories and models were sparsely adopted, such as, situated reasoning (n = 3) (Durning et al., 2011b, 2012a; McBee et al., 2017), three-track mind (n = 3) (Liu et al., 2000; Tona, 2004; Unsworth, 2001), dual processing theory (n = 2) (Durning et al., 2012b; Schaye et al., 2019), and cognitive load theory (n = 2) (Durning et al., 2012a; Farri et al., 2012). Theories mentioned by only one paper included cognitive continuum theory, pattern recognition, script theory, schemata, narrative reasoning, system thinking, problem space, naturalistic decision making, Schon’s model of reflection, therapeutic inferences, three levels of concept, and personal construct theory (Table 4).

**Table 4:** Local theories and middle-range theory cited by included studies

Dimension	Local theories	Middle-range theories
Cognition	*Pharmacy*: (Abdel-Tawab et al., 2011), (Bartels, 2013) *Medicine*: (Barrows & Feltovich, 1987), (Goldszmidt et al., 2013), (Juma & Goldszmidt, 2017), (Lamond et al., 1996) *Nursing*: (P. Benner & Wrubel, 1982), (P Benner, 1984), (Donald, 1992), (Ekman et al., 2011), (Marsha E Fonteyn, 1998), (Fowler, 1997), (Jennifer Jones, 1989), (Levett-Jones et al., 2010), (Offredy, 2002) *Physical therapy*: (Ian Edwards, 2007), (Edwards et al., 2004), (Jensen et al., 1990), (Mark Jones et al., 2008)	Information processing theory (Newell, 1972) Dual process theory (Marcum, 2012) Hypothetico-deductive reasoning (Elstein et al., 1990) Three-track mind (Fleming, 1991) Cognitive continuum theory (Hammond & Mellers, 1999) Pattern recognition (Barrows & Feltovich, 1987) Script theory (Tomkins, 1978) Problem space (Newell, 1972) Therapeutic inferences (Patel & Groen, 1986) Cognitive load theory (Paas et al., 2003) Three levels of concept (Roth & Frisby, 1986) Narrative reasoning (Ian Edwards et al., 1998)
Metacognition	(Lajoie & Lu, 2012), (Meijer et al., 2006), (Wainwright et al., 2010)	Schon's model of reflection (Schön, 1987), (Zimmerman, 2008)
Contextual	(Henderson, 2002)	System thinking/approach (Checkland, 1993) Situated reasoning (Greeno, 1989)
Ethical	(Barnitt & Partridge, 1997), (Triezenberg & Purtilo, 1996)	
Multiple aspects	(McLellan et al., 2012)	Naturalistic decision making (Klein, 2008) Personal construct theory (Kelly, 1970)

**Fig. 2:**
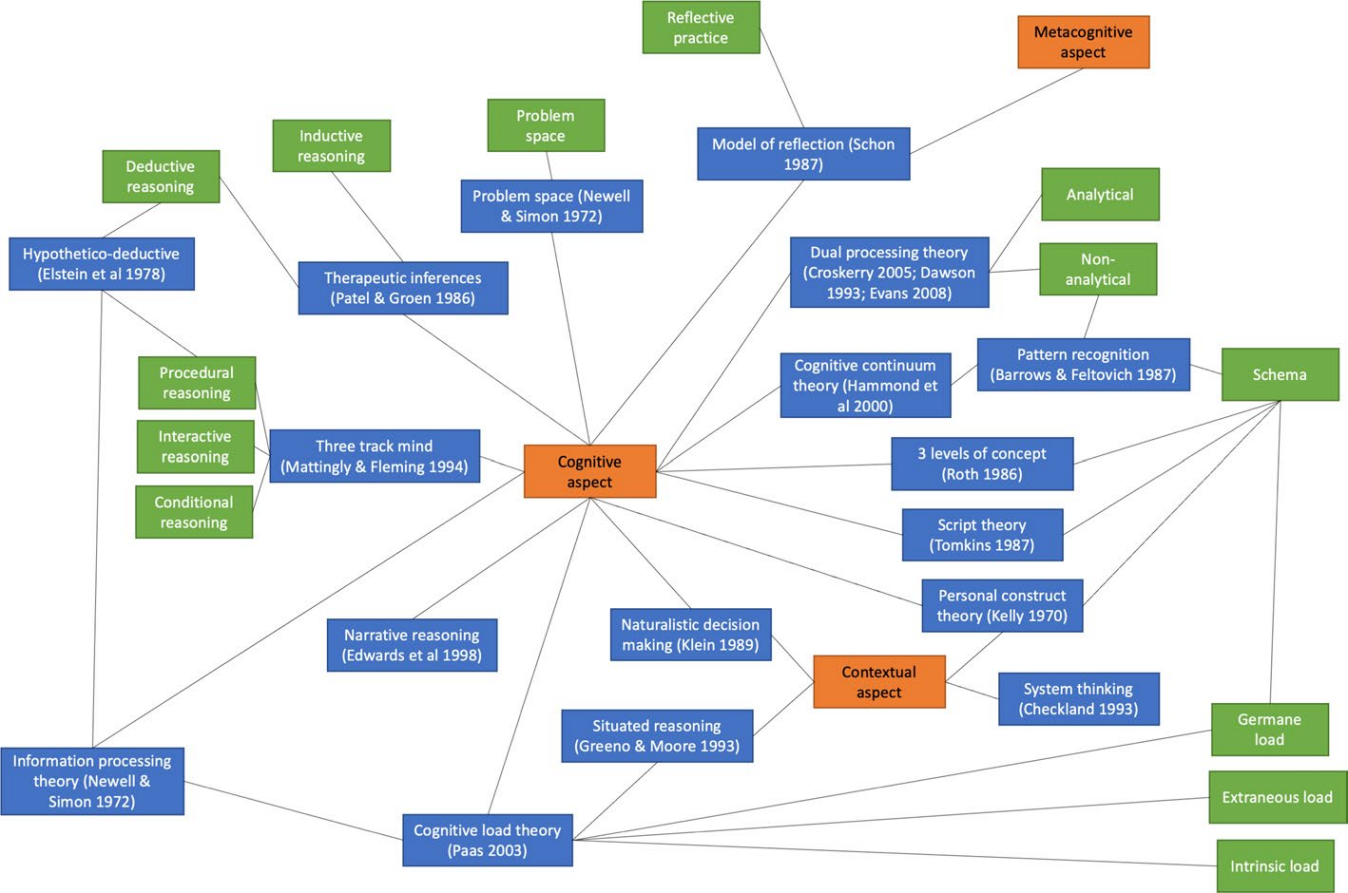
[printed in color]. The relationship between the articles underlying theoretical frameworks and their components. The lines between boxes indicate a relationship in some way. Orange: dimension of clinical decision making; Blue: theories and models; Green: concepts and components (Higgs et al., 2008)

As with many theories, a large proportion of these middle-range theories overlapped or originated from each other. Figure 2 lists the middle-range theories in three different dimensions of therapeutic reasoning and demonstrates how they are related. For example, IPT serves as a basis for the development of hypothetico-deductive reasoning (Hoffman, 2007), which is a middle-range theory related to procedural reasoning, one of the components of the three-track mind model (Higgs et al., 2008). From the contextual perspective, situated reasoning relates closely to cognitive load theory, which in turn is a theory from the cognitive perspective. Overall, there is an overwhelming number of theories related to the cognitive aspect, with some interconnections with the contextual aspect, while there is limited research conducted on metacognition or its relationship with other aspects.

Figure 2 was developed from underlying theories used by the included articles, and the connections between these theories were made if either an article reconciled two models, or two models intrinsically shared a common component. For example, Durning and colleagues discussed how situated reasoning could be related to cognitive load theory, such that contextual factors negatively affected cognitive load (Durning et al., 2012a); hypothetico-deductive reasoning contains a concept of deductive thinking, which is synonymous with the backward thinking concept in Patel & Groen’s model of therapeutic inferences (Patel & Groen, 1986). Therefore, one function of this figure is to determine the relationship between models, offshoots from a specific model, as well as the extent to which an aspect of therapeutic reasoning is being researched.

### What local theories of therapeutic reasoning do authors employ?

In contrast to middle-range theories, local theories were understandings that originated from specific investigations and only applied to therapeutic reasoning (McKenney & Reeves, 2013). In this review, authors cited (n = 19), built off (n = 5), and produced (see results) local theories. Nineteen studies cited at least one of 27 different local theories of therapeutic reasoning (Table 4). 15 of the cited local theories originated from primary research, while the remaining literature was secondary sources such as textbooks and reviews. Of the 15 studies that cited previous primary research local theories, only five studies applied these to their codebooks. Only three local theories were cited by more than one study, which are the five rights model by Levett-Jones et al. (2010), a book chapter on clinical reasoning models by Ian Edwards (2007), and a book chapter of clinical reasoning in physiotherapy by Mark Jones et al. (2008).

Similar to the middle range theories, we categorized local theories into metacognitive, contextual and cognitive aspects, but with the addition of ethical considerations. Literature focusing on cognitive tasks was the most frequently cited local theories and originated from several distinctive health professions. Out of the 19 publications, only one study cited a local theory for ethical considerations in physical therapy, two studies cited a local theory for metacognition, and one study cited a local theory for contextual factors in nursing. One cited local theory, the theoretical model of prescribing, combined more than one aspect, i.e., contextual factors and cognitive tasks, into their models.

### What are the key results of studies that investigate therapeutic reasoning?

Six key result categories were found from the content analysis of each article’s content: (1) identifying themes (n = 48), (2) characterizing and testing previous local theory (n = 44), (3) exploring factors (n = 39), (4) developing new local theory (n = 30), (5) testing tools (n = 12), and (6) testing hypothesis (n = 8).

Many articles identified discrete generalized themes of therapeutic reasoning (n = 48). Themes mentioned in these articles revolved around characteristics of the decision-maker, context, ethics, knowledge, cognitive tasks, reflection and nature of reasoning processes. For example, Gibson and colleagues compared the reasoning process of an expert and novice occupational therapist (Gibson et al., 2000). Emerging themes included definitions of clinical reasoning, sources used when reasoning, factors influencing clinical reasoning, ability to prioritize, patient viewed as an individual, patients’ role in treatment, and clinical reasoning as an evolving process. Another example, Simmons et al. (2003) described 11 different heuristics that registered nurses used to aid in retrieving information and applying knowledge. However, the outcomes of using such heuristics were not measured in this study.

39 articles explored diverse factors of the therapeutic reasoning process. Largely, factors could be external confounders, which are related to workplace context, socio-culture, economic, politic, decision-maker’s personal experience, character traits and perception. On the other hand, factors could also be an internal component of the reasoning process itself, which are the data that decision-maker analyses when performing therapeutic reasoning, such as their own knowledge, patient-related clinical information or different informative references. These factors were often identified as part of a larger thematic analysis thus can overlap and complement themes, or as a secondary purpose of more in-depth studies.

44 studies referred to previous local theories, to various degrees, as a foundation for their findings. For example, McBee et al. (2019) tested a list of clinical reasoning tasks proposed by Juma and Goldszmidt (2017). There are three subcategories: characterizing, testing and discovering alignment. 26 studies characterized previous local theories, which elaborated on their descriptions and created new perspectives by applying existing models to novel populations. Compared to characterizing, testing existing local theories involved examining them to ultimately approve or reject the applicability of the model without significant enrichment (n = 15). Discovering alignment occurred when studies identified similarities between their findings and existing local theories without using them as the underpinning framework for their definitions (n = 7). These articles found that the therapeutic reasoning patterns fitted some common middle-range theories such as hypothetico-deductive model, pattern recognition or schema.

30 articles developed a new local theory of therapeutic reasoning. Authors developed these local theories inductively or semi-inductively, often by open coding their research data. For example, Croft et al. (2018), using literature on clinical reasoning in various health disciplines, identified the core thinking processes of clinical reasoning: considering prescription in context, retrieving information, identifying medication-related issues, processing information, collaborative planning, decision making and reflection. The local theories established relationships, order or structure of therapeutic reasoning. 22 articles developed clinical decision-making models focusing on the cognitive process. The models can be demonstrated as overarching separate “subprocesses”, “phases” of reasoning, a sequence of “cognitive tasks”, a “schema” of clinical reasoning, or a framework connecting cognition to various components of decision-making such as patient-related considerations, emotions and experience. Although terminologies differed across these articles, they generally emphasized thinking processes including analysis, synthesis and evaluation. Three studies produced models that distinguished the reasoning processes of different levels of expertise (Boyer et al., 2015; Sinclair, 2010; Wainwright et al., 2010). Two other articles delineated decision typology of health professionals, which can be about the function or the nature of such decisions (Bucknall et al., 2016; Munroe, 1996). Additionally, reasoning patterns and heuristics used by nurses were depicted by two articles, both of which mentioned pattern-matching (ME Fonteyn & Grobe, 1992; Simmons et al., 2003). Lastly, two articles heavily incorporated the use of metacognition and reflection into their clinical reasoning models, particularly an osteopathic metacognitive framework and a model on how reflections inform clinical decision-making (McIntyre et al., 2018; Wainwright et al., 2010).

A few studies (n = 12) appraised a tool or instrument including decision-making guides (Baker et al., 2017), fMRI (Chang et al., 2016; Durning et al., 2012b), think-aloud (Corcoran et al., 1988), educational materials (Fiddler et al., 2016), and software (Farri et al., 2012; Satter et al., 2012). For example, Baker et al. (2017) described an application of the tool named Systematic Clinical Reasoning in Physical Therapy (SCRIPT). Results showed that SCRIPT enabled physiotherapy learners to appropriately and systematically generate hypotheses and queries, facilitating deeper investigation when the origin of the foot conditions was indefinite. Using SCRIPT, patients were successfully referred with diagnostically helpful information. Another example is from two papers examining the feasibility of functional neuroimaging fMRI in elucidating therapeutic reasoning. Chang et al. (2016) examined the differences in medical students’ brain fMRI patterns between solving a recall and a reasoning task; Durning et al. (2012b) found limited imaging evidence to support dual-processing theory and speculated that clinical decision-making may be multi-dimensional. Both studies agreed that neuroimaging might be a viable method to explore and explain therapeutic reasoning. Farri et al. (2012), framing their investigation on cognitive load theory (Paas et al., 2003), explicated clinicians’ reasoning processes when reviewing a lengthy electronic medical record, which in turn inform the recommendations for further refinement of the system to improve readability.

A small number of articles (n = 8) tested original hypotheses (Chang et al., 2016; Chaturvedi, 1994; Horsky et al., 2017; Kavanagh, 1997; McBee et al., 2017; Price et al., 2017; Sedgwick et al., 2014; Tona, 2004). These articles validated a self-generated hypothesis, and neither tested pre-existing theories nor generated hypotheses. For example, Chang et al. (2016) hypothesized that tasks requiring problem-solving would involve brain regions associated with high cognitive function. The functional neuroimaging results showed that executive functions areas were significantly utilized during problem-solving tasks, while recall tasks were associated with the activation of the amygdala, which is well-known for long-term memory modulation.

Some other articles made comparisons between different levels of expertise (Chaturvedi, 1994; MJ Curran et al., 2006a; Gibson et al., 2000; Greenwood & King, 1995; Kuiper & Pesut, 2004; Liu et al., 2000; Twycross & Powls, 2006; Unsworth, 2001; Wainwright et al., 2010; Wainwright et al., 2011). In these studies, two or more groups of healthcare participants with different lengths of practice or education were compared and contrasted to identify differences and similarities. For instance, on one hand, Price et al. (2017) hypothesized that under certain conditions, reliance on intuition would support accurate decision-making, even among novices. Although most participants relied more heavily on analysis than on intuition, the use of intuition while managing a familiar complication was associated with more accurate decision-making. On the other hand, Gibson et al. (2000) compared clinical reasoning between a novice and an experienced occupational therapist. It was evident that the novice therapist tended to rely on structured, intradisciplinary, protocol-guided processes, while the experienced therapists demonstrated a more intuitive, complex, as-required reasoning.

It is not surprising that mostly investigated was the cognitive perspectives of clinical reasoning. The ability to resolve complex problems and choose the optimal treatment is often considered to belong to the cognitive domain (Croft et al., 2018; Levett-Jones et al., 2010). It is also the cognitivistic approach that is suitable for studies aimed to compare and contrast novice and expert reasoning. Our findings are also consistent with Higgs & Jones that hypothetico-deductive reasoning is the most popular model in clinical reasoning research (Higgs et al., 2008). A few other models were also found such as pattern recognition, cognitive continuum theory and therapeutic inferences.

## Discussion

This review described articles of therapeutic reasoning processes characteristics, methods, use of theory, and key results. Additionally, to our knowledge, this is the first paper attempting to map previous therapeutic reasoning models. Overall, we found that therapeutic reasoning processes are studied by various professions including, but not limited to, medicine, nursing, physical therapy, occupational therapy. There was a diverse variety of models and theories constructed from a no less diverse methodology pool. These articles may either solely explore therapeutic reasoning as a standalone objective, or investigate therapeutic reasoning alongside other processes, for example, diagnostic reasoning. Multiple aspects of therapeutic reasoning were examined to different degrees between studies.

Contrary to Cook and colleagues’ assertion that research was limited, we sourced a total of 87 articles investigating therapeutic reasoning (Cook et al., 2019). Not only was this number considered significant in sheer quantity, but also reflected a larger underexplored body of literature about therapeutic reasoning. It was surprising that there had been no previous synthesis of these studies. We speculated that it might be challenging to formulate an effective search strategy as the terminology in clinical reasoning literature is often complex. Also, therapeutic reasoning may manifest a specific set of terminology depending on the profession (Meredith E Young et al., 2020). For example, to describe clinical reasoning that focuses on therapy, authors may use the terms “management reasoning”, “therapeutic reasoning”, “treatment decision making” or the umbrella term “clinical reasoning”, as seen particularly in nursing, occupational and physical therapy. Moreover, there are several challenges in conducting a thorough and sound scoping review in health profession education (Thomas et al., 2020). The subjectivistic nature of methodologies means that health education literature has inherently high heterogeneity (Thomas et al., 2020). Consequently, the synthesis of the evidence may result in low-quality and inappropriate inferences (Thomas et al., 2020). Although the volume of research is promising, there is a need for a more connected body of literature studying therapeutic reasoning. Future researchers should focus on studying understudied aspects while building off previous studies. As a field, more direction is needed for researchers on when to use specific theories and how to apply them.

Several implications can be drawn from our review. Firstly, we identified that the majority of articles did not cite other studies found in the search strategy. Instead, the authors of these articles constructed their methodology based on middle-range theories and utilized an inductive approach to generate new local theories (n = 28). In some cases, it was unknown which theories or studies underpinned the study (e.g., Faucher et al., 2012). This indicated a diverse, however fragment, ecosystem of therapeutic reasoning literature. Secondly, we speculated that therapeutic reasoning research is so context-driven that it is almost impractical to replicate across different populations, expertise levels and professions. For example, a model describing therapeutic reasoning in pharmacy students may not be translatable to medical residents. As a result, it is difficult to compare results across studies. Thirdly, we recognized a large number of descriptive (i.e., aim to describe and explain phenomena) and comparative (i.e., comparing between levels) studies, but not many prescriptive studies (i.e., aim to predict). Outcomes of therapeutic reasoning were also rarely assessed. Future researchers should study the connection between process and outcomes of therapeutic reasoning.

The main purpose of scoping reviews is to capture the current breadth of literature in a field, and more importantly, to identify areas for future inquiries (Arksey & O'Malley, 2005). Firstly, we recommend building a more associated ecosystem of therapeutic reasoning models. We have established a lack of cross-citation among local theories, with a majority of articles building their own model from a middle-range theory. Instead of saturating (or rather diluting) therapeutic reasoning with more models and theories, researchers are encouraged to seek, compile and synthesize available models relevant to their practice. Towards this point, this review provided the preliminary attempt to map previous models to other professions. As with any other types of research, the synthesis of past studies may yield a deeper understanding of therapeutic reasoning (McKenney & Reeves, 2013). While there might never be a universal theory, we recognized the importance of theories: theories can promote more rigorous methodology for researchers and provide a foundation for effective teaching and learning tools for educators (McKenney & Reeves, 2013; Varpio & Ellaway, 2021).

Secondly, we encourage exploring new methodological avenues for therapeutic reasoning research. To date, qualitative studies predominate the area, particularly coding transcriptions of patient encounters. Qualitative methodology has proven its usefulness and versatility in examining and characterizing therapeutic reasoning processes (El Hussein et al., 2014; Ericsson & Simon, 1980). Future researchers should consider novel methodologies to produce a more diverse set of conclusions. Overall, there are several models that characterize therapeutic reasoning processes, but it is less known which of these steps are the most important for certain situations. To ascertain which steps are most important, larger data sets and quantification are required.. Qualitative study is based on subjectivist epistemology: the same data set can be interpreted differently based on researchers’ predisposed experiences (Thomas et al., 2020). Future researchers could develop approaches that can measure therapeutic reasoning steps efficiently from large data sets. For example, researchers could employ computer science and machine learning to optimize data collection and analysis, such as voiceover (Moylan et al., 2015), auto-coding (Kaufmann et al., 2020), epistemic network analysis (Shaffer et al., 2016), and more.

Finally, we recommend future studies to not only focus on describing processes, but also on assessing the effectiveness, predicting outcomes, and evaluating maturation of therapeutic reasoning systems. Ensuring practitioners’ clinical reasoning is a crucial competency requirement and therefore should not be overlooked (Daniel et al., 2019). While many studies compared accuracy between expert and novice therapeutic reasoning, the results stopped at describing therapeutic reasoning as a series of processes and models, with the expert models often being deeper and intuitive. There was only a modest number of studies examining the development of therapeutic reasoning, i.e., the transition from “novice” to “expert” (Boyer et al., 2015; Cunningham et al., 2019; Sinclair, 2010). To address the issue, similar to assessing clinical reasoning (Daniel et al., 2019), one may use the multi-strategy approach to ensure other dimensions of therapeutic reasoning are also evaluated. For example, a reflective conversation may be integrated in a think-aloud session to discuss reasoning strategies or identify errors in clinical reasoning; here, both the think-aloud and reflection can serve as useful data for examining effectiveness of therapeutic reasoning development and reasoning itself.

Unexpectedly, while metacognition was widely acknowledged for the governing of cognitive processes, research of this domain was surprisingly lacking, both in terms of its extent of research and its relationship to cognitive models. Only Schon’s model of reflection-in-action and reflection-on-action was assessed in one study in physical therapy (Wainwright et al., 2010). Zimmerman (2008)’s compendium of self-regulated learning research was only superficially cited by one article (Lajoie & Lu, 2012). Metacognition appears to be crucial in clinical reasoning. Being aware and able to regulate cognition, namely cognitive forcing strategies, may help thinkers recognize and avoid biases in their decision-making processes (Croskerry, 2003). Unfortunately, there has been little progress in metacognition research.

There are limitations in our study. Although 87 articles in a span of three decades were covered, we may have missed some articles from other databases or in other languages, which could contribute significantly to the field. Publications in non-English speaking countries may portray a different pattern from the one we have identified, especially taking into account that therapeutic reasoning is highly context-dependent. We cannot guarantee that the corpus of articles represents a complete picture to the current state of therapeutic reasoning research. Nonetheless, we attempted to develop an initial mapping of therapeutic reasoning models and theories to capture the breadth of literature to a degree. It is also not within our scope to critically appraise each study, and as a result, the quality of these studies was not assessed.

## Conclusion

The reviewed articles suggest that therapeutic reasoning processes are of interest to various professions from students to professionals. These processes can be measured and studied through various means, but most researchers have studied them through qualitative coding procedures. The reviewed articles represent little connection between each other. Rarely do authors build off previous local theories of therapeutic reasoning. Also, there was no consistency in the use of middle range theories including hypothetico-deductive reasoning, dual-process model, or situative reasoning. We believe this field of research would benefit from greater discourse on underpinning middle-range theories and expansion of methodologies.

### Supplementary Information

Below is the link to the electronic supplementary material.

Supplementary file1 (DOCX 120 KB)
