# Supplementary material for: A scoping review of therapeutic reasoning process research
Source: Adv Health Sci Educ Theory Pract. 2023 Apr 12;28(4):1289–310. doi: 10.1007/s10459-022-10187-7 (PMC10624714; doi:10.1007/s10459-022-10187-7)
Supplement: Supplementary file 1 — Supplementary file1 (DOCX 120 KB) [file 10459_2022_10187_MOESM1_ESM.docx]

# Appendix. All included studies (n = 87)

| **Citation** | **Year** | **Profession** | **Country** | **Methodological approach** | **How therapeutic reasoning was stimulated** | **How was therapeutic reasoning measured** | **Participants** | **Theoretical framework** | **Summary of results** |
| --- | --- | --- | --- | --- | --- | --- | --- | --- | --- |
| Abuzour, A. S. (2017). An Investigation Into the Learning and Clinical Reasoning Processes of Independent Prescribers. (Ph.D.). The University of Manchester (United Kingdom), Ann Arbor. | 2017 | Pharmacy, nursing | UK | Study 1: Systematic review Study 2: Audio-diary + interview Study 3: Think aloud to explore cognitive skill | Study 2: reflection on learning experiences Study 3: clinical vignettes from validated exam scenarios | By coders (grounded theory) | Study 2: 6 pharmacists and 7 RN Study 3: 10 pharmacist and 11 nurse independent prescribers | Inductive Model of prescribing (McLellan et al 2012) | The clinical knowledge, experience, professional background, context and attitudes of independent prescribers in this study greatly influenced their clinical reasoning and decision-making. A distinct pattern was found in The process undertaken to reach A clinical decision, which is presented as A prescribing model: case familiarisation, generating initial hypotheses, case assessments, final hypothesis, decision-making  Influences on clinical reasoning were broadly categorised into themes: individual Influences, context and interactions. These themes showed that individual and socio-cultural aspects of prescribing heavily influenced The prescribers. |
| Baker, S. E., Painter, E. E., Morgan, B. C., Kaus, A. L., Petersen, E. J., Allen, C. S., . . . Jensen, G. M. (2017). Systematic Clinical Reasoning in Physical Therapy (SCRIPT): Tool for the Purposeful Practice of Clinical Reasoning in Orthopedic Manual Physical Therapy. Physical Therapy, 97(1), 61-70. | 2017 | Physical therapy | US | Filling a form on clinical reasoning in first patient visit then use the form reflect in subsequent visits | Patient encounter (mock case) | Mentors' feedback Systematic clinical reasoning in physical therapy tool (SCRIPT) | 1 mentee physical therapist | NA | SCRIPT enables appropriate hypothesis generation and guides query. SCRIPT also helps with understanding of clinical reasoning in teaching. |
| Bartels. (2013). Analysis of experienced pharmacist clinical decision-making for drug therapy management in ambulatory care setting. University of Minnesota. | 2013 | Pharmacy | US | Semi-structured interview post patient interaction | Post patient interaction | By coders (thematic analysis) | 6 pharmacists | Inductive | Experienced pharmacists use a different model (compared to other health professionals) of clinical decision-making using constant dialogue between two different types of knowledge (objective and context-related). The pharmacist must perform an active modification step necessary to combine the objective, factual information with the contextual, patient-related knowledge --> complete situational knowledge. Experienced Pharmacist Clinical Decision-Making Model was developed: objective knowledge, context-related knowledge, modification, situational understanding, inadequate  Enabling factors and barriers to clinical decision-making are unique for each context: time, disease speficic, lmited expertise |
| Blondon, K. S., Maitre, F., Muller-Juge, V., Bochatay, N., Cullati, S., Hudelson, P., . . . Nendaz, M. R. (2017). Interprofessional collaborative reasoning by residents and nurses in internal medicine: Evidence from a simulation study. Medical Teacher, 39(4), 360-367. | 2017 | Nursing and Medicine | Switzerland | Interviews: semi-structured and stimulated-recall while reviewing the videotape | Four prototypical patient cases | By coders Quantitative | 14 nurses and 14 medical residents | Inductive; Goldszmidt et al 2013 | Verified a semi-inductive model: 5 dimensions supporting collaborative reasoning by Goldszmidt  Contributing factors: task overload   Comparison between nurse and resident and as a collab team: efficiency. Residents and nurses differed in their reasoning processes |
| BOYER, L., TARDIF, J. & LEFEBVRE, H. 2015. From a Medical Problem to a Health Experience: How Nursing Students Think in Clinical Situations. Journal of Nursing Education, 54, 625-32. | 2015 | Nursing | Canada | Students' clinical judgement was assessed and presented | Narrative presented by assessors | By coders (thematic analysis + conceptual map) | Year 1-3 students | Inductive | Built a cognitive model of learning, with key learnings and indicators (3 levels) - Level 1: Intervening Step by Step  - Level 2: Investigating to Understand  - Level 3: Responding to the Whole Person  Contributed to understanding of nursing reasoning process development.  Parameters of nursing clinical judgment in undergraduate nursing education. |
| BRENNAN, D. S. & SPENCER, A. J. 2002. Factors influencing choice of dental treatment by private general practitioners. International Journal of Behavioral Medicine, 9, 94-110. | 2002 | Dentistry | Australia | Survey | Survey questions | By coders (content analysis) Quantiative (cluster analysis) | 1202 dentist | Inductive Consulted literature on factors | Identified factors that influence treatment choices: treatment constraints, periodontal status, tooth status, etc.  Classification of dentists by treatment choice responses |
| Bucknall, T. K., Forbes, H., Phillips, N. M., Hewitt, N. A., Cooper, S., Bogossian, F., & Investigators, F. A. (2016). An analysis of nursing students' decision-making in teams during simulations of acute patient deterioration. Journal of Advanced Nursing, 72(10), 2482-2494. | 2016 | Nursing | Australia | Reflection with cued recall | Simulated patient encounter | By coders (Framework Analysis approach) | 12 three-membered nursing teams | Inductive | Revelation of 11 types of decisions: information seeking; patient assessment; diagnostic; intervention/treatment; evaluation; escalation; prediction; planning; collaboration; communication and reflective.  Factors that influence decision-making: Patient distress, uncertainty and a lack of knowledge |
| Burman, M. E., Stepans, M. B., Jansa, N., & Steiner, S. (2002). How do NPs make clinical decisions? Nurse Practitioner, 27(5), 57-64. | 2002 | Nursing | US | Interviews | 2 clinical vingettes (1 acute, 1 chronic) with some basic information | By coders (grounded theory) | 36 primary care NPs | Inductive | How decision-making is influenced by 1experience and 2context: influential factors (eg family, community, patient)  Describe themes regarding the nature of reasoning: 1iterative, spiral, putting the pieces together, 2focused on the patient’s agenda, 3grounded their decision making in the patient/family and community context, .  Describe inductive model of how care planning occur: 2 distinct subprocesses in DM: Diagnostic reasoning (pattern matching) & care planning + strategies.  Validate models: Pattern-matching |
| Chaffey, L., Unsworth, C., & Fossey, E. (2010). A grounded theory of intuition among occupational therapists in mental health practice. British Journal of Occupational Therapy, 73(7), 300-308. | 2010 | Occupational therapy | Australia | Semi-structured interviews | Questions on meaning of intuition and a professional example of intuition in practice (recall) | By coders (constant comparative method) | 9 OTs with 4 months to 25 years of experience | Inductive CCT (Hammond 1996) | Themes found in responses about the meaning and use of intuition: 1Intuition is elusive and ‘underground’, 2Experience leads to a more comfortable use of intuition, 3Intuition relies on therapists understanding their own and others’ emotion, 4Intuition partners analysis, embedded within clinical reasoning.  Developed a grounded theory for intuition (although the author stated that CCT provided a framework to understand participants’ oscillation between intuition and analysis) depicting intuition as the synergy of professional experience, with the understanding and use of emotions, resulting in an oscillation between intuition and analysis within clinical reasoning. |
| CHANG, H. J., KANG, J., HAM, B. J. & LEE, Y. M. 2016. A functional neuroimaging study of the clinical reasoning of medical students. Advances in Health Sciences Education, 21, 969-982. | 2016 | Medicine | Korea | MRI during problem solving | 40 multiple choice questions | By neuroimaging | 25 medical students | NA | Compared to the recall task, the reasoning task is associated with executive function and deductive reasoning. During the recall task, significant activation was observed in the brain regions that are related to memory and emotions. Our results support that medical students mainly solve clinical questions with deductive reasoning involving prior knowledge structures and executive functions. The problem-solving questions induced the students to utilize higher cognitive functions compared with the recall questions. |
| Chaturvedi, R. K. (1994). Reasoning about therapeutic and patient management plans in respiratory medicine by physicians and medical students. (Ph.D.). McGill University (Canada), Ann Arbor | 1994 | Medicine | Canada | Think-aloud | 2 cases | By coders (framework analysis) | 16 medical staff (4 experts, 4 sub-experts, 4 residents, 4 students) | Therapeutic inferences by Patel & Groen 1986 The coding scheme is based on definitions related to medical science | Use of knowledge: 3 types: basic science, pathophysiology, clinical science. Give percentage of knowledge used by each group  Directionality: both groups demonstrated forward directionality. The novices generated rule-based prototypical textbook descriptions based on the clinical information, and the diagnosis given in the task. The experts’ therapeutic responses showed a predominance of causal-level inferences, reflecting more backward-directed inferences than novices.  The pathophysiological explanations of the disease were generated from a different knowledge source than that used to develop therapeutic decisions. |
| Corcoran, S., Narayan, S., & Moreland, H. (1988). "Thinking aloud" as a strategy to improve clinical decision making. Heart & Lung, 17(5), 463-468. | 1988 | Nursing | US | Think-aloud Transcribed patient interaction | Simulated patient interaction | By coders (Analysis of transcript to reveal themes) | 1 nurse (transcript) | Inductive | Revealed the nurse's goal, primary concern, knowledge utilization (textbook, practical, rule of thumb), and cognitive processes (hypothesis activation, hypothesis evaluation).  2 strategies in implementing TA: peer dialogue, mentoring. 3 suggesttions: (1) selecting cases, (2) prompting thinking aloud, and (3) inferring practical knowledge and cognitive processes. |
| CRISTANCHO, S., LINGARD, L., FORBES, T., OTT, M. & NOVICK, R. 2017. Putting the puzzle together: the role of 'problem definition' in complex clinical judgement. Medical Education, 51, 207-214. | 2017 | Medicine | Canada | Draw pictures  Interview to explain the meaning behind those pictures System thinking (Senge 2006) | Operation | Gallery walk cross comparison | 5 surgeons | Systems thinking & Systems approach | Challenge two assumptions from current models of clinical judgement: that experts hold a fixed and static definition of the problem and that consequently the focus of the expert’s work is on solving the problem.  Surgeons' perspectives on the definition of problem: concerns about ensuring standard of care, balancing personal emotions versus care choices, coordinating resources, maintaining control while in the midst of personality clashes. |
| Croft, Gilligan, Rasiah, Levett-Jones, Schneider (2017). Thinking in pharmacy practice: A study of community pharmacists' clinical reasoning in medication supply using the think-aloud method. Pharmacy, 6, 1:1-14 | 2017 | Pharmacy | Australia | Concurrent and retrospective think aloud | Mock case | By coders (deductive directed content analysis + inductive) | 10 pharmacists | Inductive Clinical reasoning cycle (nursing) Biopsychosocial model of clinical reasoning (physio) Literature on clinical reasoning model: Bartels et al., (2013), Levett-Jones et al., (2009), Abdel-Tawab et al., (2011), Abdel-Tawab et a., (2010), Edwards et al., (2007) | Identified seven core thinking processes used during the supply process: (1)considering prescription in context, (2)retrieving information, (3)identifying medication-related issues, (4)processing information, (5)collaborative planning, (6)decision making and (7)reflection. |
| Crowe, M. L. (2012). Allocation of health care resources at the point of care: An exploratory study of the perceptions and decision making of nurse practitioners delivering primary care services in community clinics. University of Akron | 2012 | Nursing | US | Survey (online and paper) | 85 questions (likert-scale) + 1 qualitative question | Quantitative analysis & coding (content analysis) | 68 nurses | Inductive Naturalistic decision making (Klein, 1989, 1993) Recognition Primed Decision model (2008) | “Compromised decision making” and “care impacted by the inability to pay for services” were themes emerging from the examples of the ethical issues described by nurse practitioners in narratives.  The most frequently used strategies to manage resource scarcities were substitution of another option (34%) and explanation of why the service could not be provided (44%).  Poor efficacy of a therapy (85%), small marginal benefit (78%), short life expectancy (49%), and age > 85 years (47%) were the most frequently identified criteria for not providing services.  Respondents indicated ethical dilemmas confronted most often involved (1) patients whose capacity for decision making was impaired, (2) patients for whom the preferred course of treatment was not pursued due to the patient’s insurance status, (3) patients for whom the preferred course of treatment was not pursued due to the rules for payment, and (4) scarcity of resources that required a difficult choice. |
| Cunningham, S., Litwin, B., Fernandez-Fernandez, A., & Canbek, J. (2019). Influence of residency training on the clinical reasoning development of Kenyan physiotherapists. Journal of Manual & Manipulative Therapy (Maney Publishing), 27(4), 237-244. | 2019 | Physical therapy | Kenya | Assessment of patient examination and one-on-one reflection interview post exam | Live patient cases | Clinical performance evaluation tool By coders (phenomenological approach) | 14 residents in the third cohort of the residency program (3 had not completed the modules for eligibility to sit live exam) | Hypothetico deductive reasoning Narrative reasoning Pattern recognition | Residents (n = 14) demonstrated a statistically significant improvement in their ability to perform an examination of a patient and determine a hypothetical diagnosis.  The clinical reasoning process described by the participants included the hypothetical deductive and narrative reasoning models.  The residents did not appear to incorporate pattern recognition during the patient assessment. |
| Curran, M. J., Campbell, J., & Rugg, G. (2006). An investigation into the clinical reasoning of both expert and novice podiatrists. Foot, 16(1), 28-32. | 2006 | Podiatry | UK | Think-aloud | Real patient cases selected by an author | By coders (grounded theory approach) | Study 1: 5 experts, 9 novices (year 2&3) Study 2: 3 biomechanics and 3 surgery-specialized | Inductive | Themes in clinical reasoning seen in novice and expert, generalist and specialist:  Expert generalist + Novices: (1) Visual cue, (2) touch, (3) patient questions, (4) diagnostic statement.  Specialists: (1) Visual cue, (2) touch cue, (3) patient questions, (4) diagnosis, (5) examination, (6) general statement, (7) results of examination, (8) statement by patient, (9) treatment aim.  Validate Tacit knowledge and schemata |
| Curran, M., Rugg, G., & Campbell, J. (2006b). Would podiatrists benefit from an expert system for clinical reasoning and diagnosis? A study using laddering. Foot, 16(2), 71-75. | 2006 | Podiatry | UK | Phone interview with probing questions | Questions on the decision making process | Content analysis, a hierarchical value map | 12 podiatrists | Inductive | A hierarchical map depicts podiatrists root of clinical reasoning.  Initial response values the palpation of the foot, building a picture of a foot condition and being able to use clinical reasoning frequently and immediately.  - Why 1: the value of observation of foot a condition and knowing about the problem with a foot in order to formulate a diagnosis. - Why 2: accuracy of the diagnosis was a very important value as from this the podiatrists could start to formulate a treatment plan.  Validate the theory of schemata |
| DURNING, S., ARTINO, A. R., PANGARO, L., VAN DER VLEUTEN, C. P. M. & SCHUWIRTH, L. 2011. Context and clinical reasoning: understanding the perspective of the expert's voice. Medical Education, 45, 927-938. | 2011 | Medicine | US | 1) Post-encounter form after viewing video 2) Think-aloud while viewing video | Videotapes potraying cases with different contextual factors | By coders (constant comparative approach) | 25 internists | Inductive Theoretical framework of situated cognition and ecological psychology | # Category 1: components influencing the impact of contextual factors -Theme 1: requirement for additional resources -- Sub-theme 1: germane or peripheral resource use -Theme 2: dismissing or using the contextual factor -- Sub-theme 1: credibility of the source of the contextual factor Sub-theme 2: emotions invoked by the contextual factor -- Sub-theme 3: appointment goals (pragmatic or learning orientation) - Theme 3: the influence of encounter setting  # Category 2: mechanisms for addressing contextual factors - Theme 1: balancing the goals of the patient and doctor - Theme 2: interaction of factors  # Category 3: consequences of contextual factors for patient care - Theme 1: missing of key findings - Theme 2: calibration (as beneficial to patient care or as a barrier to patient care) |
| Durning, S. J., Artino, A. R., Boulet, J. R., Dorrance, K., van der Vleuten, C., & Schuwirth, L. (2012). The impact of selected contextual factors on experts' clinical reasoning performance (does context impact clinical reasoning performance in experts?). Advances in health sciences education : theory and practice, 17(1), 65-79. | 2012 | Medicine | US | 1) Post-encounter form after viewing video 2) Think-aloud while viewing video | Videotapes potraying cases with different contextual factors | Mainly PEF were analyzed (score-based): descriptive analysis | 25 internists | Inductive Situated cognitive framework, cognitive load theory (Ericsson et al 2006) | Survey and cognitive load measures were correlated with PEF performance.  Cognitive load measures were negatively correlated with PEF scores.  The presence of selected contextual factors appeared to influence diagnostic more than therapeutic reasoning  Contextual factors appear to impact expert physician performance.  The selected contextual factors studied correlated, as predicted by CLT, with our PEF and think-aloud measures. The correlations between selected contextual factors and PEF section performance varied. |
| Durning, S. J., Graner, J., Artino, A. R., Jr., Pangaro, L. N., Beckman, T., Holmboe, E., . . . Oakes, T. (2012). Using functional neuroimaging combined with a think-aloud protocol to explore clinical reasoning expertise in internal medicine. Military Medicine, 72-78. | 2012 | Medicine | US | Combination fMRI and think-aloud | MCQ | By coders, fMRI | 17 expert internists | Dual process theory | fMRI findings provide some support for the presence of analytic and nonanalytic reasoning systems.  Statistically significant activation of prefrontal cortex distinguished answering incorrectly versus correctly  Activation of precuneus and midtemporal gyrus distinguished not guessing from guessing |
| Farri, O., Pieckiewicz, D. S., Rahman, A. S., Adam, T. J., Pakhomov, S. V., & Melton, G. B. (2012). A qualitative analysis of EHR clinical document synthesis by clinicians. AMIA ... Annual Symposium Proceedings/AMIA Symposium, 2012, 1211-1220. | 2012 | Medicine | US | Think-aloud | Viewing 9 patient EHR while performing routine clinical tasks | By coders (frameworks for protocol analysis, content analysis) | 6 clinical interns | Inductive Cognitive load theory (Paas 2003, Barnett 2004) | Interns established correlations of significance and meaning between problem, symptom and treatment concepts to inform hypotheses generation and clinical decision-making. A model of common cognitive pathways was made  Barriers in information synthesis from EHR documents include difficulty searching for patient data, poor readability, redundancy, and unfamiliar specialized terms. |
| FAUCHER, C., TARDIF, J. & CHAMBERLAND, M. 2012. Optometrists' clinical reasoning made explicit: a qualitative study. Optometry and Vision Science, 89, 1774-84. | 2012 | Optometry | Canada | Videotape of patient interaction Retrospective think aloud interview while viewing video | Examination of live patients | By coders | 4 competent and 4 expert optometrists | Literature review on clinical reasoning Inductive | 7 themes (codes): (1) Patient centeredness, (2) Planning, (3) Investigative process, (4) Analysis/reflection, (5) Mental representation of the patient’s clinical situation and refinement of this representation, (6) Diagnoses formulation, (7) Management plan.  Expert-level optometrists are more patient centered, formulates an earlier mental representation of the patient’s clinical situation (including diagnosis formulation), plans examinations more thoroughly, is able to analyze and reflect during cognitively demanding tasks, and draws up his or her care management plan throughout the entire examination. |
| Fiddler, H., Robinson, C., & Rudd, T. (2016). A case based wiki develops critical thinking skills and clinical reasoning capability in pre-placement physiotherapy undergraduate students. Physiotherapy, 102 (Supplement 1), e263-e264. | 2016 | Physiotherapy | UK | Collaborative wiki for students | Mock case | By coders | 64 physiotherapy students | Inductive | Critical thinking processes were identified in the wiki texts (number); recognise (2), understand (54), analyse (38), evaluate (60) and create (6).  Students used diagnostic reasoning 26 times, procedural reasoning 34 times, patient centred reasoning 37 times and ethical reasoning once. |
| FINCH, E., GEDDES, E. L. & LARIN, H. 2005. Ethically-based clinical decision-making in physical therapy: process and issues. Physiotherapy Theory & Practice, 21, 147-62. | 2005 | Physiotherapy | Canada | Semi-structured interview then reflection | Discussion on recent ethical conflicts | By coders (using themes from literature) | 8 physical therapists | Inductive Literature on ethics: Barnitt 1998, Triezenberg 1996 | Results: 4 categories with subordinal themes #1. The integration of ethical issues in the clinical decision-making process a. participants’ feelings and reflections about making the decision b. pressure from the patient, the family, or other HCPs c. resources used in the decision-making process  #2. Patient welfare a. treatment effectiveness b. patient autonomy c. quality of life  #3. Professional ethos of the PT (distinguishing characteristics and beliefs that guide behavior) a. professional role and boundaries b. patient advocacy c. professional collegiality  #4. Health care economics and business practices a. conflict of interest b. funding c. allocation of scarce resources d. endorsement of equipment |
| FONTEYN, M. E. & GROBE, S. J. 1992. Expert nurses' clinical reasoning under uncertainty: representation, structure, and process. Proceedings - the Annual Symposium on Computer Applications in Medical Care, 405-9. | 1992 | Nursing | US | Think-aloud then prompted retrospective recall | A mock case | By coders (protocol analysis) | 10 nurses | Inductive Information processing theory (Newell & Simon) | The concepts that were most frequently referred to by subjects were: action, amount, problem, sign, time, treatment, and value.  Identified a set of operators to explain subjects' predominant reasoning processes: study, conclude, choose, and explain.  Nurses make sense out of information from either the outside environment (providing the context of the problem-solving task) or from an inside storage structure (LTM) by forming relationships between concepts on which they are concentrating.  Several heuristics identified: pattern matching, predictive reasoning, enumerating a list, forward reasoning, identifying critical indicators, questioning. |
| Fossum, M., Alexander, G. L., Goransson, K. E., Ehnfors, M., & Ehrenberg, A. (2011). Registered nurses' thinking strategies on malnutrition and pressure ulcers in nursing homes: a scenario-based think-aloud study. Journal of Clinical Nursing, 20(17-18), 2425-2435. | 2011 | Nursing | Norway | Think-aloud interviews | 4 mock cases | By coders (deductive content analysis) | 30 nurses | 17 thinking strategies by Fonteyn (1998) | The three most commonly used thinking strategies were ‘making choices’, ‘forming relationships’ and ‘drawing conclusions’.  None of the nurses performed a structured risk assessment of malnutrition or pressure ulcers.   Registered nurses started with assessing data from the scenarios, but after a short and elementary assessment they moved directly to planning. |
| FUNKESSON, K. H., ANBACKEN, E. M. & EK, A. C. 2007. Nurses' reasoning process during care planning taking pressure ulcer prevention as an example. A think-aloud study. International Journal of Nursing Studies, 44, 1109-1119. | 2007 | Nursing | Sweden | Think-aloud session | Mock case | By coders (protocol analysis, content analysis) | 11 nurses | Cognitive operators and strategies by Fowler (1997) Inductive | Most nurses in this study had an extensive reasoning as a whole, of which the main part directly or indirectly connected to prevention of pressure ulcer.  The six most frequently used concepts (in total of 16) where sign, valuation, general action, nursing action, paramedic action and goal.  The two most frequently used were implicational and significative. Causal assertions were rare.  Cognitive strategies were infrequently used. Instead, the reasoning was dominated by the use of operators. However the operator ‘‘connecting’’ was rare. |
| GIBSON, D., VELDE, B., HOFF, T., KVASHAY, D., MANROSS, P. L. & MOREAU, V. 2000. Clinical reasoning of a novice versus an experienced occupational therapist: a qualitative study. Occupational Therapy in Health Care, 12, 15-31. | 2000 | Occupational therapy | US | Audiotaped/transcribed semi-structured interview with case study | A case study | By coders (cut and put method) | 1 novice and 1 exp therapist | Inductive | Themes which emerged include definitions of clinical reasoning, sources used when reasoning, factors influencing clinical reasoning, ability to prioritize, patient viewed as an individual, patients’ role in treatment, and clinical reasoning as an evolving process. |
| Gilliland, S. J. (2015). Believing, Thinking, and Doing: Physical Therapist Students' Clinical Reasoning and Characterizations of Practice. Believing, Thinking & Doing: Physical Therapist Students' Clinical Reasoning & Characterizations of Practice, 1-1. | 2015 | Physiotherapy | US | 1) In-depth conceptual interview 2) Observation of patient encounter, followed by post encounter form & interview | 1) Discussion of their experience with physical therapy 2) Patient encounter | By coders (structural coding) | 8 PT students | Whole thesis: Built on Edwards & Jones’ (2007) model of clinical reasoning  Code for characterizations of practice: - Dall’Alba’s (1998, 2002) - Gilliland, 2012; Gilliland & Fischer, 2014  Code for clinical reasoning: - Participant’s actions/decisions:American Physical Therapy Association, 2003 - Verbal interactions with the patient: Jensen, Shepard, and Hack 1990 - Statements of diagnostic ideas, contributing factors, and judgments as hypotheses: Barrows & Feltovich, 1987; Jones, Jensen, & Edwards, 2008. - The coding categories for the hypotheses were derived from Jones et al.’s (2008)  - Coded the reasoning strategies: Edwards et al 2004 - Explanations during the retrospective think-aloud: Schon, 1983; Wainwright et al., 2010 | The participants demonstrated four qualitatively different characterizations of physical therapy practice: Diagnosing and fixing problem, Assessing and educating the patient, Supporting the patient while diagnosing and treating, Working together with the patient.  Factors Influencing Students’ Perspectives on Practice: Experiences cited by the students  Five primary patterns of reasoning emerged including: trial and error, following protocol, the hypothetico-deductive process, reasoning about pain, and analysis of patient behavioral patterns.  The participants in this study demonstrated three patterns of reasoning errors during their encounter with the patient. These include failing to generate a key hypothesis, ruling out a hypothesis without sufficient reasoning, and hanging on to a hypothesis in the face of conflicting findings.  The participants who demonstrated reflection in action demonstrated a greater ability to adapt their examination and evaluation process to the unfolding findings.  Proposed four capacities for clinical reasoning and their interrelations. |
| GILLILAND, S. & WAINWRIGHT, S. F. 2017. Patterns of Clinical Reasoning in Physical Therapist Students. Physical Therapy, 97, 499-511. | 2017 | Physiotherapy | US | Patient review and think-aloud reflection | A simulated patient encounter | By coders (thematic analysis) | 8 physiotherapy students | Hypothetico-deductive (Jones et al 2008) Inductive | Four primary patterns of reasoning emerged: following protocol, the hypothetico-deductive process, reasoning about pain, and analysis of patient behavioral patterns.  Students formed hypotheses about the patient’s condition throughout their examination process.  2 primary patterns of reasoning errors during their encounter with the patient: failing to generate a key hypothesis and hanging on to a hypothesis in the face of conflicting findings. |
| Grace, S., Orrock, P., Vaughan, B., Blaich, R., & Coutts, R. (2016). Understanding clinical reasoning in osteopathy: A qualitative research approach. Chiropractic and Manual Therapies, 24(1). | 2016 | Osteopathy | Australia, New Zealand, UK | Focus group discussion | 2 questions on clinical reasoning | By coders (grounded theory) | 10 osteopathy educators | Inductive | 5 key themes: (1) Clinical reasoning does not lead to a single diagnosis, (2) Clinical reasoning occurs in many contexts, (3) Clinical reasoning occurs in two different stages, (4) Clinical reasoning calls on a number of metaskills, (5) Clinical reasoning in osteopathy is different from clinical reasoning in other health disciplines |
| Greenwood, J., & King, M. (1995). Some surprising similarities in the clinical reasoning of 'expert' and 'novice' orthopaedic nurses: report of a study using verbal protocols and protocol analyses. Journal of Advanced Nursing, 22(5), 907-913. | 1995 | Nursing | Australia | Concurrent and retrospective think aloud | Live patient assessment | By coders | 9 pairs of expert and novice nurses | Inductive A research technique for nursing by Jones (1989) Roth 1986 IPT (Newell & Simon) | Using the concept of "3 levels of concepts" by Roth 1986, they mapped out the use of basic concepts, subordinate concepts and superordinate concepts used by novices and experts  Expert and novice nurses share many basic concepts  Experts used more basic and subordinate concepts than novices  Experts consistently used more stratergies to manipulate the information they possessed, where as novices only outperformed in the use of 'information gathering' |
| Hagedorn, R. (1996). Clinical Decision Making in Familiar Cases: A Model of the Process and Implications for Practice. British Journal of Occupational Therapy, 59(5), 217-222. | 1996 | Occupational therapy | UK | Think-aloud and reflection | Recall of a newly referred patient | Coding and macroanalysis | 6 occupational therapists | Problem space (Newell and Simon 1972) | Therapists use schematic processing to speed the identification of problems and to indicate potential solutions and actions (rapid and automated)  Therapists used the occupational therapy process in a free and unstructured fashion in order to acquire and evaluate cues, recognise patterns, identify problems and potential solutions, manipulate information in relation to the past, the present anu the future, and make decisions concerning actions. |
| Higuchi, K. A., & Donald, J. G. (2002). Thinking processes used by nurses in clinical decision making. Journal of Nursing Education, 41(4), 145-153. | 2002 | Nursing | Canada | Narrative and clinical notes of patient care | Live patient | By coders | 8 nurses | Donald 1992a (6 major thinking processes) | 6 processes were used by nurses: Description, Selection, Representation, Inference, Synthesis, Verification  14 different operations were identified in medical nurses, and 12 were found in surgical nurses |
| HOLLINGSHEAD, N. A., MEINTS, S., MIDDLETON, S. K., FREE, C. A. & HIRSH, A. T. 2015. Examining influential factors in providers' chronic pain treatment decisions: a comparison of physicians and medical students. BMC Medical Education, 15, 164. | 2015 | Medicine | US | Questions on which information they used/would have used to make decision | Computer-simulated patients | Quantitative analysis | 35 medical students, 50 physicians | Inductive Literature based on prior research (Stutts 2010, Hirsh 2008, Hirsh 2010), clinical guidelines (Chou 2007) and experience | Most participants reported using patients’ pain histories (97.6 %) and pain description (95.3 %) when making treatment decisions,  They would have used information about patients’ previous treatments (97.6 %) and average and current pain ratings (96.5 %) had this information been available.  Compared to physicians, medical students endorsed more frequently that they would have used patients’ employment and/or disability status  A greater proportion of medical students wanted information on patients’ use of illicit drugs and alcohol to make treatment decisions; while a greater proportion of physicians reported using personal experience to inform their decisions. |
| HORSKY, J., AARTS, J., VERHEUL, L., SEGER, D. L., VAN DER SIJS, H. & BATES, D. W. 2017. Clinical reasoning in the context of active decision support during medication prescribing. International Journal of Medical Informatics, 97, 1-11. | 2017 | Medicine | US, Netherland | Think-aloud in response to a drug interaction alert | EHR with drug-drug interaction alerts (different severities) | By coders (inductive coding?) | 32 clinicians | Inductive | Participants actively looked for ways to avoid or mitigate patient risk.  The two most frequent decisions for high-severity alerts were to proceed with ordering both interacting medications and then to monitor patients for signs of adverse effects, or to lower the initial dose of one or both drugs  In responding to low-severity alerts clinicians routinely prescribed medications without explicitly controlling risks when they felt confidentthe effects were unlikely to occur for a particular patient or that they would be mild or tolerated  On many occasions, clinicians could not make a definitive decision atthe time an alert was active because they wanted to consult a pharmacist or talk to the provider who originally prescribed the interacting drug to coordinate care |
| Jenny, J., & Logan, J. (1992). Knowing the patient: one aspect of clinical knowledge. Image - the Journal of Nursing Scholarship, 24(4), 254-258. | 1992 | Nursing | Canada | Interview | Recall from experience | By coders (constant comparative method grounded theory) | 16 expert critical care nurses | Concept of "Knowing the patient" (Benner & Wrubel 1989) Inductive | Model surveys 5 variables: context, causal conditions, intervening conditions, strategies and consequences. |
| Kanters, A. E., Shubeck, S. P., Sandhu, G., Greenberg, C. C., & Dimick, J. B. (2018). Justifying our decisions about surgical technique: Evidence from coaching conversations. Surgery, 164(3), 561-565. | 2018 | Surgery/medicine | US | Coaching sessions focusing on coachee's performance | Video review of patient examination and coaching conversation | By coders (thematic) | 10 pairs of surgeons (10 expert coaches, 10 coachees) | Inductive | Three themes were identified: Reaction to negative outcome, Expert opinion, Limited supporting evidence - Reaction to negative outcome: Participants reported modifications in surgical technique after a particularly negative postoperative complication for a single patient. - Expert opinion: Participants were noted to defend the use of certain techniques or surgical decisions based on the perceived expert opinion of others. - Limited supporting evidence: Participants rarely referenced surgical literature or educational programming as the motivation for changing surgical technique. |
| Kavanagh, K. (1996). A study of clinical decision-making by expert physical therapists. | 1996 | Physical therapy | US | Think aloud and debrief questions | 2 patient encounters | Scoring instrument + ANOVA | 10 orthopedic & 10 neurologic therapists | Elstein and Bordage’s (1988) model of iterative hypothesis generation and testing, | The proportionally low frequency of Hypothesis Generation statements does not support iterative hypothesis testing as a model that best characterizes this problem solving process.  Subjects use a consistent problem solving process across Expert Therapist Groups and across Evaluation Situations, |
| KHATAMI, S., MACENTEE, M. I., PRATT, D. D. & COLLINS, J. B. 2012. Clinical reasoning in dentistry: a conceptual framework for dental education. Journal of Dental Education, 76, 1116-28. | 2012 | Dentistry | Canada | Think-alouod interview | 6 vignettes | By coders (deductively, bottom-up inductive approach) | 18 dental students | Inductive Clinical decision making by Higgs & Jones 2008 | The student participants reasoned their way through the vignettes by 1) collecting and evaluating information, 2) identifying and interpreting problems, 3) evaluating options, and 4) diagnosing diseases and planning treatment.  The students were usually guided by ritual; forward or backward reasoning or a combination of the two; pattern recognition and scripts; and decision analysis.  The students used seven reasoning strategies to interpret and address the problems: scientific, conditional, collaborative, narrative, ethical, pragmatic, and part-whole reasoning.  The students’ clinical reasoning occurred in a multilayered context including their own and their patients’ personal frames of reference. These were influenced by a larger array of social, cultural, political, and economic contexts.  The junior studentstended towards standard rituals, such as the written protocol of the clinic, whereas more experienced students were more flexible with their own routines. Senior students were more aware of their personal frame of reference, their individual view or philosophy of care, and how that influenced their interpretation of problems and their approach to care. |
| Kramer, J., Bowyer, P., O'Brien, J., Kielhofner, G., & Maziero-Barbosa, V. (2009). How interdisciplinary pediatric practitioners choose assessments. Canadian Journal of Occupational Therapy, 76(1), 56-64. | 2009 | Pediatry | US | Focus groups with semi-structured guide | Questions on decision making process | By coders (inductive analysis) | 21 practitioners | Inductive | Two primary themes emerged: “Things practitioners want to know” and “Choosing what and how to assess.” - Things practitioners want to know: About the child, About the child’s environmental context - Choosing what and how to assess: “Fitting”the child, Balancing formal and informal information, Professional context |
| Kuiper, R. A., & Pesut, D. J. (2004). Promoting cognitive and metacognitive reflective reasoning skills in nursing practice: self-regulated learning theory. Journal of Advanced Nursing, 45(4), 381-391 | 2004 | Occupational therapists | Australia | Repertory grid interviews | Interviewing questions on decision making | Grid data management software (a form of quantitative analysis) | 21 occupational therapists | Personal construct theory Inductive | 6 themes: therapy task (assessment), therapy task (upper limb), factors, problem-solving, standard practice, therapy task (therapist/client perspective)  Novice participants demonstrated statistically significant change in the structure of their clinical reasoning following exposure to the protocol: Prior to exposure, novices relied on therapy tasks, the problem‐solving process, environmental factors and standard practice to structure their reasoning. Following exposure, novices’ clinical reasoning changed to more closely reflect experts’ reasoning.  Changes in experts were not statistically significant |
| Ladyshewsky, R. K. (2002). A quasi-experimental study of the differences in performance and clinical reasoning using individual learning versus reciprocal peer coaching. Physiotherapy Theory & Practice, 18(1), 17-31. | 2002 | Physiotherapists | Australia | Observation Post-activity questionaire | Mock case | 4 evaluation instruments | 62 physiotherapy students | NA | Students in the RPC group signifcantly outperformed their peers in the individual learning group in the categories of physical examination, communication and clincal reasoning |
| LAJOIE, S. P., POITRAS, E., NAISMITH, L., GAUTHIER, G., SUMMERSIDE, C., KAZEMITABAR, M., TRESSEL, T., LEE, L. & WISEMAN, J. 2013. Modelling domain-specific self-regulatory activities in clinical reasoning. Lecture Notes in Computer Science (including subseries Lecture Notes in Artificial Intelligence and Lecture Notes in Bioinformatics). | 2013 | Medical students | America | Think aloud Logfile trace data | Mock cases | By coders | 30 medical students | Lajoie 2012; Zimmerman 2008; Meijer 2006 Inductive | Characteristics of help-seeking behaviours: requested help in later stage, they used 10% of their problem-solving behaviour on requesting help. Requesting help is more common with rare diseases, these patterns suggest that students requested consults while reasoning about the implications of a lab test towards their own hypotheses as well as gathering additional information regarding either the tests or a particular disease.  Antecedent and Consequent Activities during Help-Seeking: students engaged in orientation activities 3.2 times more often before, as opposed to after, asking for help. The most frequent skills that students demonstrated during the orienting phase were identifying important information. They were more likely to engage in planning or monitoring phase before seeking help. After requesting help, they were more likely to be in the evaluation phase |
| Lee, J. E., & Ryan-Wenger, N. (1997). The "Think Aloud" seminar for teaching clinical reasoning: a case study of a child with pharyngitis. Journal of Pediatric Health Care, 11(3), 101-110. | 1997 | Medical students | US | Think aloud | Seminar proceedings (mock case) | Faculty analysis of student performance | Medical students | NA | The students arrived at the correct diagnosis, although not by the most direct route. This is not unusual for novices, who are just learning how to cluster information to be more efficient in data-gathering and problem-solving.   The students requests for objective data from the physical examination were based on their hypotheses rather than on a systematic approach. Hypothesis generation is influenced not only by book knowledge but also by clinical experience; thus every student approaches the same case from a different perspective. |
| Liu, K. P. Y., Chan, C. C. H., & Hui-Chan, C. W. Y. (2000). Clinical reasoning and the occupational therapy curriculum. Occupational Therapy International, 7(3), 173-183. | 2000 | Occupational therapists | Hong Kong | In-depth interview, protocal analysis | Assessing a patient | By coders (thematic content analysis) | 12 occupational therapists | 3 types of clinical reasoning by Mattingly and Fleming (1994) | More experienced therapists use conditional reasoning that considers clients’ needs in their future lives whereas junior therapists use procedural reasoning that focuses on clients’ disabilities.   The analysis of the occupational therapy curriculum indicated that it prepared the students with an equal emphasis on theoretical and clinical subjects and fieldwork practice. The present curriculum was useful in providing educational preparation for novice therapists. |
| Mancuso, C. A., & Rose, D. N. (1987). A model for physicians' therapeutic decision making. Archives of Internal Medicine, 147(7), 1281-1285. | 1987 | Medicine | US | Interviews with mock cases, recorded in field notes | Hypothetical cases | Inductive analysis to identify pattern | 18 internists | Inductive | A 3-stage process that is called focal composite analysis: (1) selection of a few facts (focal points) and evaluation of each fact individually with respect to treatment options; (2) reassessment of the value of the focal points with respect to each other and unification of the case; and (3) summation of the values of the focal points to make the final decision. Using this model, the authors were able to predict 96% of treatment decisions  Wide variety of focal points. Of a total of 32 focal points chosen in three cases, only two focal points were predictors of the physicians' actual treatment choices. |
| May, S., Withers, S., Reeve, S., & Greasley, A. (2010). Limited clinical reasoning skills used by novice physiotherapists when involved in the assessment and management of patients with shoulder problems: A qualitative study. Journal of Manual and Manipulative Therapy, 18(2), 84-88. | 2010 | Physiotherapists | UK | Semi-structure interview | A mock case | By coders Framework analysis | 9 novice physical therapists | Inductive | 7 themes contained items that related to: history-taking, physical examination, investigations, diagnostic reasoning, clinical reasoning (diagnostic pathway), clinical reasoning (management pathway), and treatment options. Items mostly related to information gathering  Although there was some use of hypothetico-deductive clinical reasoning, there appeared to be limited understanding of the clinical implications of data gathered, and clinical reasoning through use of pattern recognition was minimal. |
| MCBEE, E., RATCLIFFE, T., PICHO, K., SCHUWIRTH, L., ARTINO, A. R., JR., YEPES-RIOS, A. M., MASEL, J., VAN DER VLEUTEN, C. & DURNING, S. J. 2017. Contextual factors and clinical reasoning: differences in diagnostic and therapeutic reasoning in board certified versus resident physicians. BMC Medical Education, 17, 211. | 2017 | Medicine | USA | Pre-study questionaire on self-rated sleepiness and burnouts Post-encounter form on diagnosis | 3 video review of clinical encounters | By coders Quantitative | 15 interns, 10 residents | Situated cognition | Accuracy of diagnostic and therapeutic reasoning did not differ between groups despite residents reporting significantly higher rates of sleepiness and burnout. Accuracy of diagnosis and treatment were uncorrelated. In both groups, the proportion scoring correct responses for treatment was higher than the proportion scoring correct responses for diagnosis. |
| McBee, E., Blum, C., Ratcliffe, T., Schuwirth, L., Polston, E., Artino, A. R., & Durning, S. J. (2019). Use of clinical reasoning tasks by medical students. Diagnosis, 6(2), 127-135. | 2019 | Medical students | USA | Think aloud | 3 video review of clinical encounters | By coders (constant comparative approach) | 17 medical students | Framework for clinical reasoning tasks by Goldszmidt et al 2017 | They used 15 reasoning tasks from Goldszmidt framework across all cases. The average number of tasks used in cases 1, 2, and 3 was (respectively) 5.6 (range 3–8), 5.9 (range 4–8), and 5.3 (range 3–10). The order in which medical students verbalized reasoning tasks varied and appeared purposeful but non-sequential.   Every participlant verbalized Task 1 (Identify active issues) in each of the cases. Task 7 (Determine the most likely diagnosis) and Task 8 (Identify modifiable and non-modifiable risk factors) were used by each of the 17 participants in at least one clinical case. Task 4 (Consider and prioritizedifferential diagnoses) was the most frequently uttered |
| MCINTYRE, C., LATHLEAN, J. & ESTEVES, J. E. 2018. Osteopathic clinical reasoning: An ethnographic study of perceptual diagnostic judgments, and metacognition. International Journal of Osteopathic Medicine, 28, 30-41. | 2018 | Osteopaths | UK | Autoethnography: a reflexive field diary | Treatment session | By coders (grounded theory) | 1 single osteopath | Inductive | Perceptual diagnostic judgments: osteopaths establish a multisensory construct during clinical reasoning which enables both analytic and intuitive decision-making strategies. 3 themes: ‘building a picture’; ‘mental imageryinto embodied senses’; and ‘multisensory perception enables pattern recognition and analysis’  Metacognition: constructs that fit within the already established themes of metaknowledge, meta-skills (analysing & assessing, predicting, planning) and meta-experiences. A model for meta-skills by Pesut & Herman broadly fits the study, with some differences  Metacognitive framework for osteopaths |
| Munroe, H. (1996). Clinical reasoning in community occupational therapy. British Journal of Occupational Therapy, 59(5), 196-202. | 1996 | Occupational therapists | Scotland | Field observation by researcher, written explanation by participants, then follow-up interview | Patient home visit | By coders | 30 occupational therapists | Inductive | 3 patterns of reasoning (ideal, real, intuitive) consisting of 3 elements: reflection,decision-making and reasoning  Decisions were classified as technical, procedural, or interactive. The majority of decisions are interactive. Reflection-in-action was commonplace  Interactive reasoning is influenced by complexity of client-therapist relationship and other factors eg contextual, economic, cultural... |
| Myers, J. R., Kiersma, M. E., & Plake, K. S. (2017). Assessment of student pharmacists' ethical decision-making. Currents in Pharmacy Teaching & Learning, 9(6), 996-1002. | 2017 | Pharmacists | USA | Survey | Difficult ethical situations | Descriptive analysis | 236 pharmacy students | NA | >90% of students agreed or strongly agreed that pharmacists have the right to refuse to dispense a medication for clinical reasons, while 45.3% agreed or strongly agreed that pharmacists had the right to refuse to dispense for moral or religious reasons. >20% of students were undecided about dispensing syringes without a prescription, pseudoephedrine tablets to a frequent purchaser, and lethal doses of narcotics.   In eight out of nine cases involving ethical decision-making, >70% of students perceived the decision to be somewhat problematic. |
| Navin, P. M. (1991). How experienced nurses gather and use data. UNIVERSITY OF MASSACHUSETTS | 1991 | Nurses | America | Post-observation interview | Interaction with patients | By coders | 6 experienced nurses | Inductive | 2 approaches, scanning mode and focusing mode, to gather information. Information that guided the scanning mode and the stimulus for subject to use the focusing mode were addressed. Responses subjects made to the answers to questions were also described. The key role of subjects' knowledge in the data gathering activities and decision-making was also included. A 3rd approach is context building mode: Data from the report as an information gathering activity occurring outside the two modes.  Experienced nurses used three activities for gathering information to make clinical decisions—listening or reading report, reading records, and interacting with patients. A model of decision-making was included: 5 stages |
| Nilsson, T., & Lindstrom, V. (2016). Clinical decision-making described by Swedish prehospital emergency care nurse students - An exploratory study. International Emergency Nursing, 27, 46-50. | 2016 | Nurses | Sweden | Written reflection on patient encounters | Patient encounters | By coders (content analysis) | 12 nursing students | Inductive | 4 themes emerged as crucial for the students’ decision-making: knowing the patient,the context-situation awareness in the ambulance service, collaboration, and evaluation. |
| O'Neill, E. S. (1999). Strengthening clinical reasoning in graduate nursing students. Nurse Educator, 24(2), 11-15. | 1999 | Nurses | America | Written journal reflecting on clinical decisions | Recall to clinical decisions, interaction with patients, think-aloud with colleague | By coders | 36 nursing students | Inductive | 5 themes: knowledge representation, knowledge use, contextual features, decision supports, and self-validation. |
| ORME, L. & MAGGS, C. 1993. Decision-making in clinical practice: how do expert nurses, midwives and health visitors make decisions? Nurse Education Today, 13, 270-6. | 1993 | Nurses, midwives, health visitors | UK | Group discussion and debate | Discussion on decision-making process | Analysed by researchers, set in context of critical literature review | 12 expert clinicians | Inductive Set in context of literature review | Factors enhancing CDM (categorised into 3 themes preparation, process, support): philosophy of care, in-depth professional knowledge, reflecting, environment, acknowledgement of intuition...   Characteristics of expert decision-maker: confident, able to solve dilemmas, flexible....  Stages in CDM: 7 broad stages, starting with establishing the philosophy of care and ending with reflecting on outcome & CDM |
| Pomeroy, S. E., & Cant, R. P. (2010). General practitioners' decision to refer patients to dietitians: insight into the clinical reasoning process. Australian Journal of Primary Health, 16(2), 147-153. | 2010 | General practitioners | Australia | Study 1: interview Study 2: quantitative cross-sectional survey | Whether to refer a patient to a dietician | Study 1: by coders (to identify themes) Study 2: descriptive statistics | Study 1: 30 GPs Study 2: 248 GPs | Inductive | 4 themes to explain GPs’ reasoning: (i) synthesising management information; (ii) forecasting outcomes; (iii) planning management; (iv) actioning the referral. A framework for thinking through and analysing the contextual factors that influencing the clinical decision making was mapped.   A concept map was developed to explain doctors’ clinical reasoning for referral, which relies on a doctor’s medical knowledge and clinical experience, the patient’s nutrition profile and a nutrition history from other health services providers. |
| Price, A., Zulkosky, K., White, K., & Pretz, J. (2017). Accuracy of intuition in clinical decision-making among novice clinicians. Journal of Advanced Nursing, 73(5), 1147-1157. | 2017 | Nurses | USA | Survey (quantitative) | Simulation scenario | Evaluation rubric + statistical analysis | 126 nursing students | NA | Reliance on intuition did not vary by familiarity of the complication. Reliance on Intuition among those in the primary nurse role was greater than those in other roles, but only on the novel complication.  Although most participants relied more heavily on analysis than on intuition, use of intuition during the familiar complication was associated with more accurate decision-making, particularly in guiding attention to relevant cues.With the novel complication, use of intuition appeared to hamper decisionmaking, particularly for those in an observer role. Correlation between role and accuracy of intuition showed mixed evidence |
| Raptis, S., Chen, J. N., Saposnik, F., Pelyavskyy, R., Liuni, A., & Saposnik, G. (2017). Aversion to ambiguity and willingness to take risks affect therapeutic decisions in managing atrial fibrillation for stroke prevention: Results of a pilot study in family physicians. Patient Preference and Adherence, 11, 1533-1539. | 2017 | Medicine | Canada | Survey (quantitative) | Case vignettes included in the surveys | Descriptive analysis | 73 family physicians | NA | 67.1% of physicians selected the correct therapeutic options in two or more of the three simulated case vignettes.   Aversion to ambiguity was associated with appropriate change to anticoagulation therapy in the management of AF (significant)  Physicians’ willingness to take individual risk in multiple domains was associated with lower errors (significant) |
| Rivett, D. A., & Higgs, J. (1997). Hypothesis generation in the clinical reasoning behavior of manual therapists. Journal of Physical Therapy Education (American Physical Therapy Association, Education Section), 11(1), 40-45. | 1997 | Physical therapists | Australia | Think-aloud | Watching a videotape of an experienced manipulative therapist and imagining that they are the therapist | By coders | 19 physical therapists working in manual therapy | Clinical reasoning model for physical therapists by Jones et al. (1992) Hypothetico-deductive models | all experts + less experts showed evidence of hypothesis generation, nearly 75% did so in the first minute.   identified 7 categories of hypothesis (same as those from Jones et al.,)  support the use of hypothetico-deductive model |
| ROOTS, S. A., NIVEN, E. & MORAN, R. W. 2016. Osteopaths' clinical reasoning during consultation with patients experiencing acute low back pain: A qualitative case study approach. International Journal of Osteopathic Medicine, 19, 20-34. | 2016 | Osteopaths | New Zealand | Video-assisted commentary | Consultations with patients | By coders (thematic analysis + content analysis) | 3 osteopaths | Inductive | 3 key themes 1) Implicit cognitive evaluations not apparent to an external observer; 2) Iterative processing of cues assembled through clinical interactions; 3) Collaborative interaction between patient and practitioner.  Each theme was presented in relation to four clinical phases (a) pre face-to-face, and face-to-face, (b) structured case history, (c) structured examination, (d) combination phase. Showed a schematic diagram of the initial diagnostic phases in an osteopathic consultation.  The existing clinical reasoning strategies of pattern recognition, hypothetico-deductive and collaborative reasoning were identified. Gave an example of consultation mapping of 1 participant |
| Satter, R. M., Cohen, T., Ortiz, P., Kahol, K., Mackenzie, J., Olson, C., . . . Patel, V. L. (2012). Avatar-based simulation in the evaluation of diagnosis and management of mental health disorders in primary care. Journal of Biomedical Informatics, 45(6), 1137-1150. | 2012 | Psychiatrist | USA | Think aloud | Virtual patients, case scenario (text-based or digitally) | By coders (propositional analysis) Assessment by DSM5 | 14 psychiatrists | Inductive | Comparing 3 groups (PCP digital, PCP text-based and expert psychiatrists) 1) Process of comprehension: identify propositions recalled or inferred to - compare the propositions by PCPs to that of domain experts: some correlation.  - some propositions were ignored by PCPs: greatest discrepancies are recognition of psychomotor agitation, retardation...for case 1, recognition of traumatic events... for case 2 2) Diagnostic decision making  - Avatar group: higher diagnostic accuracy compared to text group 3) Management plan findings - Both PCP groups were broad in their recommendation, few commonalities, considerably shorter than what is recommended and less specificity. PCPs suggested interventions that were briefly mentioned and were not explained in detail |
| Schaye, V., Eliasz, K. L., Janjigian, M., & Stern, D. T. (2019). Theory-guided teaching: Implementation of a clinical reasoning curriculum in residents. Medical Teacher. | 2019 | Medicine residents | America | 1) Quetionaires 2) Post-survey | 1) Questions about flexibility in thinking and degree of knowledge 2) Describing a scenario where they applied the principles taught | Diagnostic Thinking Inventory By coding | 71 first year medicine residents | Dual process theory (DPT) (Croskerry 2005) Script theory (ST) (Lubarsky et al. 2015). | Results: comparing 3 groups (no intervention, partial and full)  - Significant difference between groups in application of concepts  - Significant difference in describing cases in problem representation format  - No significant difference in change in DTI scores |
| SEDGWICK, M. G., GRIGG, L. & DERSCH, S. 2014. Deepening the quality of clinical reasoning and decision-making in rural hospital nursing practice. Rural & Remote Health, 14, 2858. | 2014 | Nurses | Canada | Semi-structured interviews | Scenario | Nosich’s standards of reasoning | 15 nurses | Benner (2001) | The ability to engage in deep clinical reasoning varied considerably among participants. 3 assumptions being addressed 1) nurses with more experience display greater ability to engage in clinical reasoning. But analysis showed number of practicing years were not linked to CDM 2) clinical reasoning and decision making would be a function of the interpersonal characteristics of rural nursing practice. participants with <5 years of practice demonstrated that their relationship with more experienced healthcare professionals influenced their CDM 3) being able to engage in deep CDM was dependent on being able to think creatively. Participants indicated that the need to be creative in problem solving was necessary for the provision of care. Deep CDM is a function of reflection and self-correction that requires a critical self-awareness. |
| Shilling, H. N. (2012). How Integrative and Eclectic Therapists Make Treatment Selection Decisions: A Qualitative Study. (Ph.D.). The University of Wisconsin - Madison, Ann Arbor. | 2012 | Psychologist | America | A series of 3 semi-structured interviews | A case study | By coders | 5 Integrative or Eclectic (I/E) psychologists | Inductive | 1) Treatment decisions emerged from I/E orientations: What this orientation meant to therapists, why it was chosen, how it developed.  2) I/E therapists used stable theoretical or philosophical cores, plus ample flexibility to inform treatment. Participants reported similar core approaches: humanistic, relational, dynamic, or interpersonal. Flexibility was fundamental to I/E treatment selection.  3) The therapeutic relationship was inextricably linked to the treatment selection process, impacting it in complex and subtle ways. 4) Therapists’ conflicted disavowal of Empirically Supported Treatments (ESTs) led them to feel like a silent majority.  5) Therapists based treatment selection on certain concrete, specific variables: timing, diagnosis, formal assessment, treatment goals, and larger sociopolitical contexts. However, these were less salient than other factors.  6) Therapists’ treatment decision processes were experienced as primarily implicit |
| Shubin, P. S. (1999). Therapeutic decision making of primary care nurse practitioners. (M.S.). University of Wyoming, Ann Arbor. | 1999 | Nurse Practitioner | USA | Interview | 2 vignettes | Constant comparative method By coders | 7 primary care nurse practitioners | Inductive | Themes: (a) getting to know the patient globally, (b) search for markers or clues, (c) identifying a threshold, (d) arming the patient with tools for self care, (e) looking for treatment options, (f) using resource networks, (g) putting it all together, and (h) ongoing monitoring. |
| Simmons, B., Lanuza, D., Fonteyn, M., Hicks, F., & Holm, K. (2003). Clinical Reasoning in Experienced Nurses. Western Journal of Nursing Research, 25(6), 701–719. https://doi.org/10.1177/0193945903253092 | 2003 | Nurses | America | Think aloud | Assessment of patient | By coders (protocol analysis) | 15 nurses | Inductive Information processing theory (Newell & Simon) | Concepts to concentrate on while they were reasoning: amount; care provider; condition; day, time, and date; device; diagnosis; event; family; frequency; location; missing clinical data; patient; plan; rationale; status; test; treatment; and value.  4 types of assertions (information linking) were found: (a) anticipative, (b) causal, (c) declarative and (d) evaluative. 3 concepts were used in forming assertions: test, treatment, and problem   5 reasoning processes: describe, explain, plan, evaluate, and conclude.  11 heuristics: drawing conclusions, recognizing a pattern, forming relationships... |
| SINCLAIR, K. 2003. A model for the development of clinical reasoning in occupational therapy. Hong Kong Polytechnic (People's Republic of China). | 2003 | Occupational therapist | Hong Kong | Think aloud interviews | Client interaction | Constant comparative method By coders | 30 occupational therapy students | Inductive Dreyfus & Dreyfus | Skill acquisition is a continuum. Big jumps between competent and proficient levels. Competent tends to rely on protocol and not so confident about their thinking. Experts tends to rely on intuition and no longer follows rules.  Challenges: nature of assignments, communication, understanding the role of OT  Experts organize information in schemata, easily accessible - SOLO taxonomy. Created a grading scheme using the SOLO. |
| Sinopoulou, V., Summerfield, P., & Rutter, P. (2017). A qualitative study of community pharmacists’ clinical decision-making skills. International Journal of Pharmacy Practice, 25(S1), 4-39. | 2017 | Pharmacists | UK | Think aloud during (students) Observation during and thinkaloud after (clinicians) | Discussion on decision-making process | By coders (Framework analysis) | 8 pharmacists | Inductive | Themes: poor diagnostic knowledge, ability of the pharmacists and a non-evidence based approach to decision-making.   Their motives within consultations is establishing an appropriate product to recommend, rather than the need to establish a diagnosis  Aware of limitations of the methods they used the need to ‘delve deeper’ during consultations |
| Slater, M. C. (1999). Clinical decision-making in baccalaureate nursing students: A qualitative field study. University of Pennsylvania, | 1999 | Nurses | USA | Phase 1&2: focus group interview Phase 3: observation | Phase 1&2: Discussion on experience and perceptions of decision making Phase 3: Patient care | By coders (Constant comparative method) | 28 nursing students | Inductive | 7 themes of student nurse clinical decision-making : the Process of Clinical Decision-making; "Knowing"-Knowledge; "Doing"-Experience; The Clinical Instructor, Classroom Instruction, Staff Relations and Being a Student + subthemes  3 categories of process in student nurse clinical decision-making: problem oriented, validating- checking in, priority setting + subthemes => model of clinical decision making process |
| Smith, M., Higgs, J., & Ellis, E. (2008). Characteristics and processes of physiotherapy clinical decision making: a study of acute care cardiorespiratory physiotherapy. Physiotherapy Research International, 13(4), 209-222. | 2008 | Physiotherapist | Australia | Semi-structured interviews | Episode of patient care | By coders | 14 cardiorespiratory physiotherapists | Inductive | CDM is focussed on decisions about: patient's problems, intervention, and evaluation of effectiveness. The process of making decisions varied according to the focus of the decision.   Characterize the process of each type of decision, incl a model of decision-making processes + factors in choosing an intervention  Identify attributes of acute care cardiorespiratory physiotherapy clinical decisions  CDM involved complex reasoning processes that were cyclic, evolving and flexible in nature, with inter-dependence and inter-relation between the different foci of clinical decision making.   CDM is a social and collaborative process. |
| SOCKOLOW, P. S., YANG, Y., BASS, E. J., BOWLES, K. H., HOLMBERG, A. & SHERYL, P. 2017. Data Visualization of Home Care Admission Nurses' Decision-Making. AMIA ... Annual Symposium Proceedings/AMIA Symposium, 2017, 1597-1606. | 2017 | Nurses | USA | Focus groups + follow-up phone calls | Case study | By coders (thematic content analysis) | 6 registered nurses | Inductive | 7 themes of information needed for CDM: patient, resource external, home environment...   5 themes related to decision: Visit timing & frequency, Plan of Care, Medication Reconcilliation and Other services  Node-link analysis characterizes relationship between tasks, information & decisions |
| THACKRAY, D. & ROBERTS, L. 2017. Exploring the clinical decision-making used by experienced cardiorespiratory physiotherapists: A mixed method qualitative design of simulation, video recording and think aloud techniques. Nurse Education Today, 49, 96-105. | 2017 | Physiotherapist | UK | Think-aloud then interview while reviewing videotape | Case scenario | By coders (content analysis + thematic analysis and framework approach) | 8 cardiorespiratory physiotherapists | Inductive Five-rights clinical reasoning (Levett-Jones 2010) The collaborative hypothetico-deductive (Jones et al., 2000) | CDM allign with: Five-rights clinical reasoning (Levett-Jones 2010) and The collaborative hypothetico-deductive (Jones et al., 2000). CDM demonstrated by the physiotherapists was complex, interactive and iterative.   Information processing occurred continuously throughout the whole interaction with the patient  Describe sequence of clinical events (9 stages)  Cognitive skills: recognition, matching, discriminating, relating, inferring, synthesising and prediction  A new conceptual model of clinical decision-making in cardiorespiratory physiotherapy. |
| THOMPSON, S., MOORLEY, C. & BARRATT, J. 2017. A comparative study on the clinical decision-making processes of nurse practitioners vs. medical doctors using scenarios in a secondary care environment. Journal of Advanced Nursing, 73, 1097-1110. | 2017 | Nurses | UK | Think-aloud and semi-structed interviews | 5 different scenarios | By coders (protocol analysis) | 10 NPs 5 MDs | Diagnostic reasoning (Elstein et al., 1978) Therapeutic reasoning (Offredy 2002) | 9 components of clinical reasoning (adapted from literature): cue acquisition, hypothesis generation, cue interpretation, hypothesis evaluation, diagnosis, treatment, advice, further advice, referral. 2 themes emerge: diagnostic and therapeutic   2 groups (doing 2 different cases) differed in total number of"cue acquisitions"  nurses take 3 minutes longer compared to doctors, but all made correct diagnosis and management  describe directly the process: eg "NP3 did not elucidate the following symptoms of the patient’s personal history or concerns that would be considered relevant for history taking", "NP4 confirmed the (field) notes by gaining rapport and questioning the cause for unprotected sex" |
| TICHELAAR, J., RICHIR, M. C., AVIS, H. J., SCHOLTEN, H. J., ANTONINI, N. F. & DE VRIES, T. P. 2010. Do medical students copy the drug treatment choices of their teachers or do they think for themselves? European Journal of Clinical Pharmacology, 66, 407-12. | 2010 | Physicians | Netherlands | Questionaire of deciding factors which influenced the decision | Written cases on what to prescribe | Quatitative analysis (One-way ANOVA) | 32 medical students, 29 general practitioners, 26 lung specialists, 24 orthopaedic surgeons, 24 internists | Inductive Literature on drug-choice related factors: Denig et al., (1992, 1993, 1994, 1988), Janknegt et al., (1997), Wierenga et al., (1989) | Students based their drug choice on the factors‘effectiveness of the drugs’ and ‘examples from medical teachers’ while clinical teachers based their drug choice on the factors ‘clinical experience’,, ‘effectiveness of the drugs’, ‘side effects of the drugs’, ‘standard treatment guidelines’, and ‘scientific literature’. |
| Tona, J. T. (2003). Variations in clinical reasoning among occupational therapy practitioners. State University of New York at Buffalo | 2003 | Occupational therapist | USA | Quantitative (scoring) assessment | Case studies | Rubric: Assessment of Reasoning ARC Statistical analysis | 72 occupational therapists | Three-track mind (Fleming, 1991) Levels of clinical reasoning (Slater & Cohn, 1991; Dreyfus & Dreyfus, 1986) | 1) Support the "three track mind": nearly all participants could be coded as procedural, interactive or conditional reasoning.Participants specializing in mental health used more interactive and conditional reasoning than those specializing in adult physical disabilities and pediatrics 2) Support the progression in 5-stage mode. Occupational therapists scored higher than students 3 & 4) The number of fragments produced in the most familiar case was different among participants in different specializations, but was not different among participants based on amount of experience.  5) No correlation. Level of reasoning was related to amount of experience and familiarity with a case, while type of reasoning was related to the type of specialization. 6) These findings were not the same in familiar and unfamiliar cases, indicating that type and level of reasoning likely do not transfer from familiar to unfamiliar situations |
| Tschikota, S. (1993). The clinical decision-making processes of student nurses. Journal of Nursing Education, 32(9), 389-398. | 1993 | Nurses | USA | Think-aloud | A simulated client situation | By coders (content analysis) + quantitative analysis | 19 female senior nursing students | Inductive Information processing theory (Newell & Simon) | 6 decision-making elements: cue, hypothesis, knowledge base, nursing interventions, search, assumption. Internal locus control nurses verbalize more elements  8 decisision-making processes: stimulus-response, listing, review/summarizing, non-decision structuring, hypothesize and test, hypothesize and treat, cascading and sequential combination. The frequency and types of process differ between internals and externals. Internals use more complex decision-making processes |
| Tulisiak, A. K., Klein, J. A., Harris, E., Luft, M. J., Schroeder, H. K., Mossman, S. A., . . . Strawn, J. R. (2017). Antidepressant Prescribing by Pediatricians: A Mixed-Methods Analysis. Current Problems in Pediatric & Adolescent Health Care, 47(1), 15-24. | 2017 | Pediatricians | USA | Semi-structured interviews | 3 clinical vignettes | By coders (thematic analysis) + quantiative analysis | 14 pediatricians | Inductive | 9 key factors associated with prescribing: functional impairment, symptom severity, age, availability of non-pharmacologic treatment...  4 key factors influencing choice of antidepressant: reported comfort with the medication, financial factors and access,  evidence, pragmatic factors  factors influencing prescribing to youths with anxiety disorders: severel themes eg: sequential treatment with psychotherapy...  parental and familiy factors: Most pediatricians considered this. Key themes were parental ambivalence, functional impairment within the family and the child's psychosocial milieu.  perceptions about safety: all believed that anti-depressants were safe for youth |
| Twycross, A., & Powls, L. (2006). How do children's nurses make clinical decisions? Two preliminary studies. Journal of Clinical Nursing, 15(10), 1324-1335. | 2006 | Nurses | Scotland | Think-aloud | 3 devised clinical scenarios | By coders | 12 nurses from surgical ward 15 nurses from medical ward | Hypothetico-deductive (analytical) model of decision-making (Lamond et al. 1996). (coding framework) | both novice and expert use hypothetico-deductive model and backward reasoning  give the type of information used (drug kardex, observations, communication with parents/child/other healthcare professionals). Experts and novices used the same information |
| UNSWORTH, C. A. 2001. The clinical reasoning of novice and expert occupational therapists. Scandinavian Journal of Occupational Therapy, 8, 163-173. | 2001 | Occupational therapist | Australia | Semi-structured interviews while viewing videotape | 3 therapy sessions | By coders & quantitative analysis | 3 experts and 2 novice occupational therapists | Mattingly & Fleming's reasoning categories (1994) | Quantitative: difference in amounts and types of clinical decision making between novice and expert eg: experts have higher frequency of clinical reasoning, experts use more ‘‘procedural and interactive’’ and ‘‘interactive and conditional’’ reasoning...  Qualitative: themes underlying the difference in the style of reasoning between novice and expert eg: therapy in flow, experience, confidence, client-centre practice, multiple actions and activities, accepting limitations |
| WAINWRIGHT, S. F., SHEPARD, K. F., HARMAN, L. B. & STEPHENS, J. 2010. Novice and experienced physical therapist clinicians: a comparison of how reflection is used to inform the clinical decision-making process. Physical Therapy, 90, 75-88. | 2010 | Physical therapists | USA | Semi-structured interviews of each pair, think aloud | Discussion, mock cases | By coders (grounded theory) | 3 pairs of physical therapists | Schon’s model of reflective practice Inductive | 3 different types of reflection: reflection-in-action (RIA), reflection-on-specific action (ROSA), reflection-on-professional-experience (ROPE). Experts use ROSA and ROPE with greater frequency, and were distinguished by use of RIA and self-assessment during interaction  a revised conceptual framework: the use of reflection to inform the clinical decision-making process |
| WAINWRIGHT, S. F., SHEPARD, K. F., HARMAN, L. B. & STEPHENS, J. 2011. Factors that influence the clinical decision making of novice and experienced physical therapists. Physical Therapy, 91, 87-101. | 2011 | Physical therapists | USA | Semi-structured interviews of each pair, think aloud | Discussion, mock cases | By coders (grounded theory) | 3 pairs of physical therapists | Inductive | 4 emerging themes: prior professional experience, sources of information, reflection, personal experience  a model comparing directive and informative factors in decision making (novice uses informative while expert directive) [in discussion]  a revised conceptual framework for clinical decision making and reflection in patient management [in discussion] |
| Weiss, M. C., & Scott, D. (2000). Clinical decision making - An application of judgment analysis and its potential for pharmacy. International Journal of Pharmacy Practice, 8(1), 33-41. | 2000 | General practitioners | England | Questionaire (both qualitative and quantitative) | Written cases | Descriptive and analytical statistics | 48 GPs | NA Literature on clinical factors: Centor RM et al., (1981), Johannessen et al., (1990), Talley et al., (1987)  Literature on social factors: Stephenson et al., (1988), Jones et al., (1990), Schindler et al., (1991) | 42% GPs incorporated social factors  Decision models relating social and clinical factors to GP's perception of sore throat severity (high fever, tonsillar exudates, swollen lymph nodes, patient's distress...) and the likelihood of dyspepsia diagnosis (pain at night, pain relieved by food, age, patient demand...) |
| White, M. R., Braund, H., Howes, D., Egan, R., Gegenfurtner, A., van Merrienboer, J. J. G., & Szulewski, A. (2018). Getting Inside the Expert's Head: An Analysis of Physician Cognitive Processes During Trauma Resuscitations. Annals of Emergency Medicine, 72(3), 289-298. | 2018 | Medicine | Canada | Cognitive task analysis: a cued-recall protocol, augmented by viewing eye-tracking video | 10 trauma cases collected during a 15-month period | By coders (thematic approach) | 4 local experts in trauma resuscitation | Inductive | identified 5 themes of macrocognitive logistic awareness, managing uncertainty, visual fixation behaviors, selective attendance to information, and anticipatory behaviors) |
| Wihlborg, J., Edgren, G., Johansson, A., Sivberg, B., & Gummesson, C. (2019). Using the case method to explore characteristics of the clinical reasoning process among ambulance nurse students and professionals. Nurse Education in Practice, 35, 48-54. | 2019 | Nurses | Sweden | Case discussion groups | Mock cases | By coders Mapping sentence + facet theory analysis | 49 nurses | Inductive Clinical reasoning process/5 rights (Levett-Jones et al., 2010) Literature on nursing aspects: Elkman 2011, Henderson 2002 | Professional experiences and reflectivity seemed to influence both the content and the process of clinical reasoning. At initiation of specialist education, more analytical reasoning was used, while the specialist nurses mainly used a non-analytical approach. Specialist nurses incorporated a larger variety of content during their reasoning.  Mapping result: showing patterns of switching between different clinical reasoning processes of each group S1, S2 & P eg: S1 & S2 both analytical and non-analytical were used while group P predominantly used non-analytical |

[1-87]

# References (all 87 articles)

1. Abuzour A. An investigation into the learning and clinical reasoning processes of independent prescribers. 2016.

2. Baker SE, Painter EE, Morgan BC, Kaus AL, Petersen EJ, Allen CS, et al. Systematic clinical reasoning in physical therapy (SCRIPT): tool for the purposeful practice of clinical reasoning in orthopedic manual physical therapy. Physical Therapy. 2017;97(1):61-70.

3. Bartels CE. Analysis of experienced pharmacist clinical decision-making for drug therapy management in the ambulatory care setting. 2013.

4. Blondon K, Maître F, Muller-Juge V, Bochatay N, Cullati S, Hudelson P, et al. Interprofessional collaborative reasoning by residents and nurses in internal medicine: Evidence from a simulation study. Medical Teacher. 2017;39(4):360-7.

5. Boyer L, Tardif J, Lefebvre H. From a medical problem to a health experience: How nursing students think in clinical situations. Journal of Nursing Education. 2015;54(11):625-32.

6. Brennan DS, Spencer AJ. Factors influencing choice of dental treatment by private general practitioners. International journal of behavioral medicine. 2002;9(2):94-110.

7. Bucknall TK, Forbes H, Phillips NM, Hewitt NA, Cooper S, Bogossian F, et al. An analysis of nursing students’ decision‐making in teams during simulations of acute patient deterioration. Journal of advanced nursing. 2016;72(10):2482-94.

8. Burman ME, Stepans MB, Jansa N, Steiner S. How do NPs make clinical decisions? The Nurse Practitioner. 2002;27(5):57-64.

9. Chaffey L, Unsworth C, Fossey E. A grounded theory of intuition among occupational therapists in mental health practice. British Journal of Occupational Therapy. 2010;73(7):300-8.

10. Chang H-J, Kang J, Ham B-J, Lee Y-M. A functional neuroimaging study of the clinical reasoning of medical students. Advances in Health Sciences Education. 2016;21(5):969-82.

11. Chaturvedi RK. Reasoning about Therapeutic and Patient Management Plans in Respiratory Medicine by Physicians & Medical Students: McGill University Libraries; 1994.

12. Corcoran S, Narayan S, Moreland H. "Thinking aloud" as a strategy to improve clinical decision making. Heart & lung: the journal of critical care. 1988;17(5):463.

13. Cristancho S, Lingard L, Forbes T, Ott M, Novick R. Putting the puzzle together: the role of ‘problem definition’ in complex clinical judgement. Medical education. 2017;51(2):207-14.

14. Croft H, Gilligan C, Rasiah R, Levett-Jones T, Schneider J. Thinking in pharmacy practice: a study of community pharmacists’ clinical reasoning in medication supply using the think-aloud method. Pharmacy. 2018;6(1):1.

15. Crowe ML. Allocation of health care resources at the point of care: an exploratory study of the perceptions and decision making of Nurse Practitioners delivering primary care services in community clinics: University of Akron; 2012.

16. Cunningham S, Litwin B, Fernandez-Fernandez A, Canbek J. Influence of residency training on the clinical reasoning development of Kenyan physiotherapists. Journal of Manual & Manipulative Therapy. 2019;27(4):237-44.

17. Curran M, Campbell J, Rugg G. An investigation into the clinical reasoning of both expert and novice podiatrists. The Foot. 2006;16(1):28-32.

18. Curran M, Rugg G, Campbell J. Would podiatrists benefit from an expert system for clinical reasoning and diagnosis?: A study using laddering. The Foot. 2006;16(2):71-5.

19. Durning S, Artino Jr AR, Pangaro L, van der Vleuten CP, Schuwirth L. Context and clinical reasoning: understanding the perspective of the expert’s voice. Medical education. 2011;45(9):927-38.

20. Durning SJ, Artino AR, Boulet JR, Dorrance K, van der Vleuten C, Schuwirth L. The impact of selected contextual factors on experts’ clinical reasoning performance (does context impact clinical reasoning performance in experts?). Advances in health sciences education. 2012;17(1):65-79.

21. Durning SJ, Graner J, Artino Jr AR, Pangaro LN, Beckman T, Holmboe E, et al. Using functional neuroimaging combined with a think-aloud protocol to explore clinical reasoning expertise in internal medicine. Military Medicine. 2012;177(suppl_9):72-8.

22. Farri O, Pieckiewicz DS, Rahman AS, Adam TJ, Pakhomov SV, Melton GB, editors. A qualitative analysis of EHR clinical document synthesis by clinicians. AMIA Annual Symposium Proceedings; 2012: American Medical Informatics Association.

23. Faucher C, Tardif J, Chamberland M. Optometrists’ clinical reasoning made explicit: a qualitative study. Optometry and Vision Science. 2012;89(12):1774-84.

24. Fiddler H, Robinson C, Rudd T. A case based wiki develops critical thinking skills and clinical reasoning capability in pre-placement physiotherapy undergraduate students. Physiotherapy. 2016;102:e263-e4.

25. Finch E, Geddes EL, Larin H. Ethically-based clinical decision-making in physical therapy: process and issues. Physiotherapy Theory and Practice. 2005;21(3):147-62.

26. Fonteyn M, Grobe S, editors. Expert nurses' clinical reasoning under uncertainty: representation, structure, and process. Proceedings of the Annual Symposium on Computer Application in Medical Care; 1992: American Medical Informatics Association.

27. Fossum M, Alexander GL, Göransson KE, Ehnfors M, Ehrenberg A. Registered nurses’ thinking strategies on malnutrition and pressure ulcers in nursing homes: a scenario‐based think‐aloud study. Journal of clinical nursing. 2011;20(17‐18):2425-35.

28. Funkesson KH, Anbäcken E-M, Ek A-C. Nurses’ reasoning process during care planning taking pressure ulcer prevention as an example. A think-aloud study. International journal of nursing studies. 2007;44(7):1109-19.

29. Gibson D, Velde B, Hoff T, Kvashay D, Manross PL, Moreau V. Clinical reasoning of a novice versus an experienced occupational therapist: A qualitative study. Occupational Therapy in Health Care. 2000;12(4):15-31.

30. Gilliland S, Wainwright SF. Patterns of clinical reasoning in physical therapist students. Physical therapy. 2017;97(5):499-511.

31. Gilliland SJ. Believing, Thinking, and Doing: Physical Therapist Students’ Clinical Reasoning and Characterizations of Practice: UC Irvine; 2015.

32. Grace S, Orrock P, Vaughan B, Blaich R, Coutts R. Understanding clinical reasoning in osteopathy: a qualitative research approach. Chiropractic & manual therapies. 2016;24(1):1-10.

33. Greenwood J, King M. Some surprising similarities in the clinical reasoning of ‘expert’ and ‘novice’ orthopaedic nurses: report of a study using verbal protocols and protocol analyses. Journal of Advanced Nursing. 1995;22(5):907-13.

34. Hagedorn R. Clinical decision making in familiar cases: A model of the process and implications for practice. British Journal of Occupational Therapy. 1996;59(5):217-22.

35. Higuchi KAS, Donald JG. Thinking processes used by nurses in clinical decision making. Journal of Nursing Education. 2002;41(4):145-53.

36. Hollingshead NA, Meints S, Middleton SK, Free CA, Hirsh AT. Examining influential factors in providers’ chronic pain treatment decisions: a comparison of physicians and medical students. BMC medical education. 2015;15(1):164.

37. Horsky J, Aarts J, Verheul L, Seger DL, van Der Sijs H, Bates DW. Clinical reasoning in the context of active decision support during medication prescribing. International Journal of Medical Informatics. 2017;97:1-11. 10.1016/j.ijmedinf.2016.09.004

38. Jenny J, Logan J. Knowing the patient: one aspect of clinical knowledge. Image--the journal of nursing scholarship. 1992;24(4):254.

39. Kanters AE, Shubeck SP, Sandhu G, Greenberg CC, Dimick JB. Justifying our decisions about surgical technique: Evidence from coaching conversations. Surgery. 2018;164(3):561-5. 10.1016/j.surg.2018.04.033

40. Kavanagh K. A study of clinical decision-making by expert physical therapists. 1997.

41. Khatami S, MacEntee MI, Pratt DD, Collins JB. Clinical reasoning in dentistry: a conceptual framework for dental education. J Dent Educ. 2012;76(9):1116-28.

42. Kramer J, Bowyer P, O'Brien J, Kielhofner G, Maziero-Barbosa V. How Interdisciplinary Pediatric Practitioners Choose Assessments. Canadian Journal of Occupational Therapy. 2009;76(1):56-64. 10.1177/000841740907600114

43. Kuiper RA, Pesut DJ. Promoting cognitive and metacognitive reflective reasoning skills in nursing practice: self-regulated learning theory. Journal of Advanced Nursing. 2004;45(4):381-91. 10.1046/j.1365-2648.2003.02921.x

44. Ladyshewsky RK. A quasi-experimental study of the differences in performance and clinical reasoning using individual learning versus reciprocal peer coaching. Physiotherapy Theory and Practice. 2002;18(1):17-31. 10.1080/095939802753570666

45. Lajoie SP, Poitras E, Naismith L, Gauthier G, Summerside C, Kazemitabar M, et al., editors. Modelling Domain-Specific Self-regulatory Activities in Clinical Reasoning. Artificial Intelligence in Education; 2013 2013//; Berlin, Heidelberg: Springer Berlin Heidelberg.

46. Lee JEM, Ryan-Wenger N. The “Think Aloud” seminar for teaching clinical reasoning: a case study of a child with pharyngitis. Journal of Pediatric Health Care. 1997;11(3):101-10.

47. Liu KP, Chan CC, Hui‐Chan CW. Clinical reasoning and the occupational therapy curriculum. Occupational Therapy International. 2000;7(3):173-83.

48. Mancuso CA, Rose DN. A model for physicians' therapeutic decision making. Archives of internal medicine. 1987;147(7):1281-5.

49. May S, Withers S, Reeve S, Greasley A. Limited clinical reasoning skills used by novice physiotherapists when involved in the assessment and management of patients with shoulder problems: a qualitative study. Journal of Manual & Manipulative Therapy. 2010;18(2):84-8.

50. McBee E, Blum C, Ratcliffe T, Schuwirth L, Polston E, Artino AR, et al. Use of clinical reasoning tasks by medical students. Diagnosis. 2019;6(2):127-35.

51. McBee E, Ratcliffe T, Picho K, Schuwirth L, Artino AR, Yepes-Rios AM, et al. Contextual factors and clinical reasoning: differences in diagnostic and therapeutic reasoning in board certified versus resident physicians. BMC medical education. 2017;17(1):211.

52. McIntyre C, Lathlean J, Esteves JE. Osteopathic clinical reasoning: An ethnographic study of perceptual diagnostic judgments, and metacognition. International Journal of Osteopathic Medicine. 2018;28:30-41.

53. Munroe H. Clinical reasoning in community occupational therapy. British Journal of Occupational Therapy. 1996;59(5):196-202.

54. Myers JR, Kiersma ME, Plake KS. Assessment of student pharmacists’ ethical decision-making. Currents in Pharmacy Teaching and Learning. 2017;9(6):996-1002.

55. Navin PM. How experienced nurses gather and use data. 1991.

56. Nilsson T, Lindström V. Clinical decision-making described by Swedish prehospital emergency care nurse students–An exploratory study. International emergency nursing. 2016;27:46-50.

57. O'Neill ES. Strengthening clinical reasoning in graduate nursing students. Nurse Educator. 1999;24(2):11-5.

58. Orme L, Maggs C. Decision-making in clinical practice: how do expert nurses, midwives and health visitors make decisions? Nurse Education Today. 1993;13(4):270-6.

59. Pomeroy SE, Cant RP. General practitioners’ decision to refer patients to dietitians: insight into the clinical reasoning process. Australian Journal of Primary Health. 2010;16(2):147-53.

60. Price A, Zulkosky K, White K, Pretz J. Accuracy of intuition in clinical decision‐making among novice clinicians. Journal of advanced nursing. 2017;73(5):1147-57.

61. Raptis S, Chen JN, Saposnik F, Pelyavskyy R, Liuni A, Saposnik G. Aversion to ambiguity and willingness to take risks affect therapeutic decisions in managing atrial fibrillation for stroke prevention: results of a pilot study in family physicians. Patient preference and adherence. 2017;11:1533.

62. Rivett DA, Higgs J. Hypothesis generation in the clinical reasoning behavior of manual therapists. Journal of Physical Therapy Education. 1997;11(1):40-5.

63. Roots SA, Niven E, Moran RW. Osteopaths' clinical reasoning during consultation with patients experiencing acute low back pain: A qualitative case study approach. International Journal of Osteopathic Medicine. 2016;19:20-34.

64. Satter RM, Cohen T, Ortiz P, Kahol K, Mackenzie J, Olson C, et al. Avatar-based simulation in the evaluation of diagnosis and management of mental health disorders in primary care. Journal of Biomedical Informatics. 2012;45(6):1137-50.

65. Schaye V, Eliasz KL, Janjigian M, Stern DT. Theory-guided teaching: implementation of a clinical reasoning curriculum in residents. Medical teacher. 2019;41(10):1192-9.

66. Sedgwick M, Grigg L, Dersch S. Deepening the quality of clinical reasoning and decision-making in rural hospital nursing practice. Rural & Remote Health. 2014;14(3).

67. Shilling HN. How integrative and eclectic therapists make treatment selection decisions: a qualitative study: The University of Wisconsin-Madison; 2012.

68. Shubin PS. Therapeutic decision making of primary care nurse practitioners: University of Wyoming; 1999.

69. Simmons B, Lanuza D, Fonteyn M, Hicks F, Holm K. Clinical reasoning in experienced nurses. Western Journal of Nursing Research. 2003;25(6):701-19.

70. Sinclair K. A model for the development of clinical reasoning in occupational therapy. 2010.

71. Sinopoulou V, Summerfield P, Rutter P. A qualitative study of community pharmacists’ clinical decision-making skills. International Journal of Pharmacy Practice. 2017;25(S1):4-39.

72. Slater DY, Cohn ES. Staff development through analysis of practice. American Journal of Occupational Therapy. 1991;45(11):1038-44.

73. Smith M, Higgs J, Ellis E. Characteristics and processes of physiotherapy clinical decision making: a study of acute care cardiorespiratory physiotherapy. Physiotherapy Research International. 2008;13(4):209-22.

74. Sockolow PS, Yang Y, Bass EJ, Bowles KH, Holmberg A, Sheryl P, editors. Data visualization of home care admission nurses’ decision-making. AMIA Annual Symposium Proceedings; 2017: American Medical Informatics Association.

75. Thackray D, Roberts L. Exploring the clinical decision-making used by experienced cardiorespiratory physiotherapists: A mixed method qualitative design of simulation, video recording and think aloud techniques. Nurse education today. 2017;49:96-105.

76. Thompson S, Moorley C, Barratt J. A comparative study on the clinical decision‐making processes of nurse practitioners vs. medical doctors using scenarios in a secondary care environment. Journal of advanced nursing. 2017;73(5):1097-110.

77. Tichelaar J, Richir M, Avis H, Scholten H, Antonini N, De Vries TP. Do medical students copy the drug treatment choices of their teachers or do they think for themselves? European journal of clinical pharmacology. 2010;66(4):407-12.

78. Tona JT. Variations in clinical reasoning among occupational therapy practitioners. 2004.

79. Tschikota S. The clinical decision-making processes of student nurses. Journal of Nursing Education. 1993;32(9):389-98.

80. Tulisiak AK, Klein JA, Harris E, Luft MJ, Schroeder HK, Mossman SA, et al. Antidepressant prescribing by pediatricians: a mixed-methods analysis. Current problems in pediatric and adolescent health care. 2017;47(1):15-24.

81. Twycross A, Powls L. How do children's nurses make clinical decisions? Two preliminary studies. Journal of Clinical Nursing. 2006;15(10):1324-35.

82. Unsworth CA. The clinical reasoning of novice and expert occupational therapists. Scandinavian Journal of Occupational Therapy. 2001;8(4):163-73.

83. Wainwright SF, Shepard KF, Harman LB, Stephens J. Novice and Experienced Physical Therapist Clinicians: A Comparison of How Reflection Is Used to Inform the Clinical Decision-Making Process. Physical Therapy. 2010;90(1):75-88. 10.2522/ptj.20090077

84. Wainwright SF, Shepard KF, Harman LB, Stephens J. Factors that influence the clinical decision making of novice and experienced physical therapists. Physical Therapy. 2011;91(1):87-101.

85. WEISS MC, SCOTT D. Clinical decision making—an application of judgment analysis and its potential for pharmacy. International Journal of Pharmacy Practice. 2000;8(1):33-41.

86. White MR, Braund H, Howes D, Egan R, Gegenfurtner A, van Merrienboer JJ, et al. Getting inside the expert’s head: an analysis of physician cognitive processes during trauma resuscitations. Annals of emergency medicine. 2018;72(3):289-98.

87. Wihlborg J, Edgren G, Johansson A, Sivberg B, Gummesson C. Using the case method to explore characteristics of the clinical reasoning process among ambulance nurse students and professionals. Nurse Education in Practice. 2019;35:48-54.
